# Nanoscale Imaging and Measurements of Grain Boundary Thermal Resistance in Ceramics with Scanning Thermal Wave Microscopy

**DOI:** 10.1021/acsami.4c08085

**Published:** 2024-08-05

**Authors:** Denis Alikin, Maria J. Pereira, Alexander Abramov, Elena Pashnina, Maria Chuvakova, Nickolay V. Lavrik, Wenjie Xie, Anke Weidenkaff, Andrei L. Kholkin, Andrei Kovalevsky, Alexander Tselev

**Affiliations:** † Department of Physics & CICECO−Aveiro Institute of Materials, University of Aveiro, Aveiro 3810-193, Portugal; ‡ School of Natural Sciences and Mathematics, Ural Federal University, Ekaterinburg 620000, Russia; § Center for Nanophase Materials Sciences, Oak Ridge National Laboratory, Oak Ridge, Tennessee 37831, United States; ∥ Materials and Resources, Department of Materials and Earth Sciences, Technical University of Darmstadt, Darmstadt 64287, Germany; ⊥ Fraunhofer Research Institution for Materials Recycling and Resource Strategies IWKS, Alzenau 63755, Germany; # Department of Materials and Ceramic Engineering & CICECO − Aveiro Institute of Materials, University of Aveiro, Aveiro 3810-193, Portugal

**Keywords:** thermal resistance, grain boundary, ceramics, scanning thermal-wave microscopy, scanning thermal microscopy

## Abstract

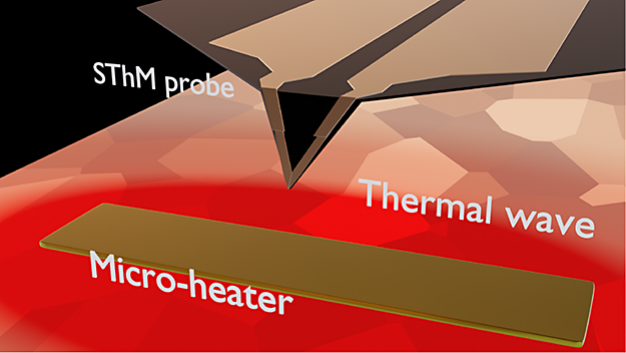

Material thermal conductivity is a key factor in various applications, from thermal management to energy harvesting. With microstructure engineering being a widely used method for customizing material properties, including thermal properties, understanding and controlling the role of extended phonon-scattering defects, like grain boundaries, is crucial for efficient material design. However, systematic studies are still lacking primarily due to limited tools. In this study, we demonstrate an approach for measuring grain boundary thermal resistance by probing the propagation of thermal waves across grain boundaries with a temperature-sensitive scanning probe. The method, implemented with a spatial resolution of about 100 nm on finely grained Nb-substituted SrTiO_3_ ceramics, achieves a detectability of about 2 × 10^–8^ K m^2^ W^–1^, suitable for chalcogenide-based thermoelectrics. The measurements indicated that the thermal resistance of the majority of grain boundaries in the STiO_3_ ceramics is below this value. While there are challenges in improving sensitivity, considering spatial resolution and the amount of material involved in the detection, the sensitivity of the scanning probe method is comparable to that of optical thermoreflectance techniques, and the method opens up an avenue to characterize thermal resistance at the level of single grain boundaries and domain walls in a spectrum of microstructured materials.

## 1 Introduction

Microstructure engineering is a commonly employed strategy for customizing and adjusting the properties of functional materials, a principle that extends to thermal properties. Material thermal conductivity plays a pivotal role in various applications, from thermal management to thermal energy harvesting, where the preservation or reduction of thermal gradients is essential. The introduction of extended phonon-scattering defects, such as grain boundaries, into materials results in a decreased thermal conduction, especially when the grain size is scaled down to the nanoscale. This reduction can be advantageous for the performance of thermoelectrics or thermal-barrier materials.^1−6^ Conversely, defects may prove detrimental in heat-sinking components within thermal management systems.^7^ Furthermore, the manipulation of ferroelectric and ferroelastic domain walls, achievable through mechanical stress or the application of an electric field, may offer a means to control heat conduction and heat flow as a new paradigm of energy regulation.^8−16^

For an efficient material design for the applications, it is of key importance to understand, quantify, and predict the role that such defects play in heat transport at the nanoscale. However, systematic studies of what mechanisms control the heat flow across grain and domain boundaries are lacking owing to the lack of tools capable of accessing temperature fields in the vicinity of individual boundaries, at the nanometer scale. As a consequence, most measurements to date have been focused on effective thermal conductivity and its dependence on the grain size or the number/density of domain walls in bulk.^11,15,17−21^ Such an approach yields assessments of only averaged boundary resistance over a large set of boundaries in a sample. The thermal resistance of individual grain boundaries could be accessed only with artificial bulk bicrystals of some materials systems.^22,23^

On the other hand, numerically simulated grain boundary resistance values are up to 1 order of magnitude lower than the experimentally obtained ones, e.g., for ceria and silicon.^23,24^ The discrepancy can be ascribed to structural nonidealities, strain, and impurities in grain boundaries. Grain boundaries, even in pure materials, are complex systems with a large parameter space. Molecular dynamics simulations, e.g., see ref (25), revealed that small distortions to local atomic environments are sufficient to dramatically reduce overall thermal conductivity across grain boundaries. Hence, the variability of the boundary resistance can be as broad as possible defects variants. The boundary resistance is sensitive, in particular, to details of the boundary atomic structure, density of contacting atoms, complete and dangling bonds, strain and empty volume, besides the properties of the grain lattice. Impurities affect resistance as well, being able both to reduce and increase the heat transfer across the boundary.^25^

Recently, spatially resolved thermoreflectance was employed for thermal imaging and measurements of grain boundary thermal resistance in ceria ceramics,^26^ polycrystalline diamond,^7^ and in a polycrystalline thermoelectric SnTe.^27^ The thermoreflectance-based techniques are optical, noncontact, pump–probe methods, where pump (heat sourcing) and probe (temperature sensing) laser spots can coincide or can be placed at different locations over a sample. The laser spot size can be as small as ∼1 μm, and the techniques were used to map thermal conductivity^7,27^ or oscillations of the temperature field^26^ in the vicinity of grain boundaries in the polycrystalline materials. These measurements, achieved with a micrometer-scale spatial resolution, revealed thermal conductivity suppression near grain boundaries^7,27^ and correlation of grain boundary thermal resistance with the grain misorientation angle.^26,27^ They represent significant progress in characterizing the heat flow both at and across individual defect entities.

The micrometer-scale spatial resolution of the optical techniques dictates a relatively large grain size of a few tens of micrometers. The large grain size is associated with more complete sintering of ceramics and, hence, with grain boundaries possessing lower energies. Nanostructuring with grain size reduction is expected to result in a larger range of grain boundary structures and resistances due to, for example, lower sintering temperatures. Therefore, such materials need specific and tailored measurements, and a smaller grain size calls for a higher spatial resolution.

In this study, we propose and test an approach for measuring grain boundary thermal resistance by probing the propagation of thermal waves across grain boundaries with a temperature-sensitive scanning probe. Previously, a thermal-wave approach was used by Hua et al.^26^ with a pump–probe optical technique for measurements of grain boundary resistance in coarse-grained cerium oxide ceramics. Hua et al. heated a sample locally by an amplitude-modulated laser beam (pump), and the thermal wave field was probed through thermoreflectance of a transducer metal layer deposited on top of the ceramic sample. The probing laser beam was focused on the sample surface and scanned through a grain boundary to measure the thermal wave phase as a function of distance across the boundary. To extract the value of the grain boundary resistance, an analytical model was fitted to the measurement data. Unlike optical techniques, where spatial resolution is limited by light diffraction effects, the spatial resolution in the scanning probe microscopy (SPM) measurements is determined by the size of the probe-sample contact and can be below 100 nm.

The method implemented in this work is an embodiment of Scanning Thermal Wave Microscopy (STWM), which is a mode of the Scanning Thermal Microscopy (SThM) family. The principles of STWM and its experimental validation were described by Kwon et al. in ref (28). In this technique, the amplitude and phase of an oscillating temperature field—thermal wave—are mapped as a function of coordinates using a temperature-sensitive probe, which is scanned over a sample. In our setup, thermal waves are generated by a harmonic electric current passing through a narrow metal strip lithographically manufactured on the sample surface. The wave amplitude and phase experience a discontinuity (jump) at a grain boundary, and the values of the jumps are used to determine the grain boundary resistance.

We performed STWM experiments on finely grained ceramics of Nb-substituted SrTiO_3_ (Nb:STO) with an average grain size of about 5 μm. Strontium titanate SrTiO_3_ (STO) exemplifies a compound often used as a model in the development of oxide-based thermoelectric materials. The grain refinement in STO ceramics was earlier demonstrated as a route to thermal conductivity reduction.^18,20^ SPM allows highly localized point-by-point measurements and, therefore, the thermal fields can be probed in the immediate vicinity of grain boundaries, which simplifies analysis of the measurement data. Here, we used this as an advantage to extract values of the grain boundary resistance. For that, we developed a simple analytical model as the first-order approximation and then used numerical simulations to find corrections to the results of the analytical model. We have achieved a detectability limit for values of the grain boundary thermal resistance of about 2 × 10^–8^ K m^2^ W^–1^, making the approach directly applicable to chalcogenide-based thermoelectrics, where typical grain boundary thermal resistances are in the 10^–8^ K m^2^ W^–1^ range.^20,27^ Characterization of the grain boundaries in oxides, where the typical grain boundary thermal resistance falls within the range of a few 10^–9^ K m^2^ W^–1^, ref (20) (although larger values were also reported^26^ requires a higher detectability. We identified that the obstacle to a higher detectability is a high sensitivity of the probe SThM response to variation of the probe-sample contact thermal resistance, which stems from a relatively low thermal resistance between the probe and environment combined with a large thermal resistance of the probe-sample contact. The effects of varying probe-sample contact thermal resistance can be offset with advanced measurement schemes. Still, with the account of spatial resolution and the amount of material participating in the detection, the performance of the scanning probe method in the current embodiment with respect to sensitivity is at least comparable with that of optical methods.

## 2 Theoretical Background of the Measurements

For a homogeneous isotropic solid whose thermal conductivity is independent of the temperature, Θ, the heat conduction equation is
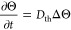
1where Δ is the Laplace operator, *D*_*th*_ is thermal diffusivity of the material:
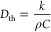
2

*k* is the thermal conductivity, ρ is the density, and *C* is the specific heat capacity. In the case of steady periodic oscillations of the temperature field in time *t* with an angular frequency ω:
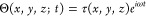
3solutions of eq 1 take forms resembling waves, which are called thermal waves. In eq 3, *x*, *y*, and *z* are Cartesian coordinates, and . As thermal waves are a consequence of the Fourier law and eq 1, they are diffusion waves, and as such, their propagation only partially resembles the propagation of ordinary waves. In particular, diffusion waves do not transfer energy,^29,30^ and while coherency of field oscillations takes a role in the distribution of the oscillation amplitude and phase, the concepts of wave reflection and interference are not applicable to the solutions of eq 1 like in the case of ordinary waves.^29,31^ The fundamental reason for that, as explained in detail by Mandelis,^31^ is that eq 1 yields infinite speed of propagation of perturbations in space (see, e.g., ref (32)). Still, while accounting for the limitations, diffusion waves can be treated mathematically like ordinary in many important situations, which will be used below. With this treatment, the waves are very highly damped, minimizing problems of multiple scattering and resonances,^33^ which means that only the wave propagating from the source needs to be taken into consideration in a media with multiple discontinuities like a polycrystal.

**Figure 1:**
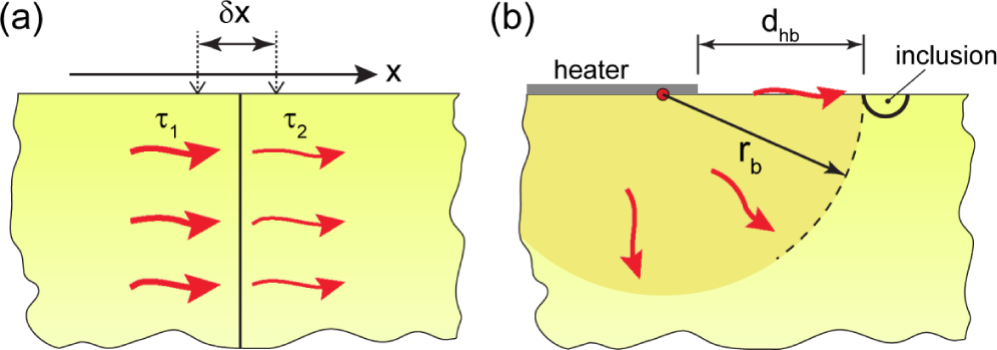
(a) Schematic of semi-infinite medium with a vertical internal boundary, where a plane thermal wave propagates along the surface. (b) Schematic of semi-infinite medium with a semispherical inclusion (grain) at the surface. The inclusion is of the same material as the matrix and is separated from the matrix by a boundary. A strip heater is on the surface of the medium. The thermal wave generated by the heater is approximated as a cylindrical wave generated by a line source on the medium surface.

The simplest model for a grain boundary in a thermal wave field is shown in Figure 1a. The medium containing the grain boundary is semi-infinite with a planar boundary plane normal to the surface. A thermal wave is a plane wave propagating along the surface and along the normal to the grain boundary. The temperature field in the plane wave is described by the equation:
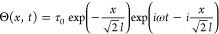
4

Here *x* is the Cartesian coordinate in the direction of the wave propagation, τ_0_ is the amplitude at *x* = 0, and:
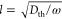
5

As seen in eq 4, a natural quantity in the description of plane thermal waves is the thermal diffusion length . In turn, we call *l thermal penetration depth*. (Note that in literature, *L*_th_ is frequently referred to as thermal penetration length.) The amplitude of the plane wave exponentially decays with distance from the source with a decay length equal to the thermal diffusion length, *L*_th_. The phase-based wavelength of the plane wave is *λ*_*th*_ = 2 *πL*_th_ ≈ 8.9*l*.

In the presence of a discontinuity, such as a grain boundary, the thermal energy is accumulated on the source side of the discontinuity and depleted on the opposite side. This resembles the reflection and transmission of ordinary waves at a discontinuity. Accumulation of heat and transmission through the boundary can be calculated using the approach outlined by Carslaw and Jaeger (with a reference to Marcus) in ref (34). This approach is based on a formal analogy between thermal wave propagation and the propagation of electromagnetic waves in transmission lines and implies that the behavior of a thermal wave can be described by methods developed for electromagnetic transmission lines. By analogy with the transmission lines, a characteristic thermal wave impedance of a medium is introduced, which is defined as^31,34^
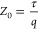
6where τ is the complex amplitude of temperature oscillations at a point in the medium, and *q* is the complex amplitude of the heat flux density at the same point (introduced by analogy with the electric current density). The characteristic impedance is a property of the medium, and it is
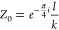
7

The effective reflection coefficient, Γ, from a grain boundary with the thermal resistance *R*_gb_ is^31,35^
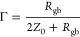
8and the effective transmission coefficient, *T*, is, consequently:
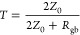
9

In an experiment, we will measure amplitudes of the temperature oscillations on both sides of the boundary. Let:
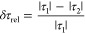
10where indexes 1 and 2 refer to different sides of the boundary, where side 1 contains the incident wave. If the measurement points are in the immediate vicinity of the boundary, the relative amplitude change is
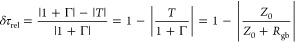
11

In turn, for the difference between oscillation phases φ = arg(τ) on the sides of the grain boundary in the boundary vicinity, δφ = φ_2_ – φ_1_, we have
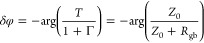
12

Note that the temperature oscillation amplitudes in the expression eq 10 are in a ratio, and hence, raw probe signals that are proportional to the amplitude of temperature oscillations can be directly used to determine *R*_gb_. All factors that are needed to convert the probe response oscillations and corresponding output signals into sample temperature (these factors can be obtained, in principle, via probe and circuit calibration) are canceled out. Furthermore, probe-sample boundary thermal resistance does not need to be known as well; however, it needs to be identical at the points on the sample that are used for the measurements.

## 3 Results and Discussion

### 3.1 Ceramic Samples and Measurement Setup

#### 3.1.1 STNO Sample

For the proof-of-concept and test measurement, we selected Nb:STO ceramic samples with a nominal composition SrTi_0.9_Nb_0.1_O_3_ (STNO). STNO is a prototype thermoelectric material.^36^ In the Nb:STO system, while A-site cation deficiency was found to enhance thermoelectric performance through synergistic improvements in charge carrier mobility and phonon scattering, the stoichiometric composition is considered more suitable as a model system for the SWTM. This approach avoids the potential risks of rutile segregation, which could introduce additional effects on thermal imaging. The sample synthesis is described in the Methods section.

Most grains in the STNO samples were 3–10 μm in size (see Figure S1a for scanning electron microscopy (SEM) image of a fractured ceramic sample). In the literature on characterization of nanograined, nanostructured STO ceramics, a noticeable reduction of thermal conductivity was reported only in fine-grained ceramics with grain size of a few tens of nanometers,^18,20^ which points to a relatively small contribution of grain boundaries in the thermal conductivity of our ceramics with 3–10 μm grains. Therefore, we utilized bulk thermal property characterization to derive material parameters for assessing grain boundary thermal resistance through localized measurements. The properties defining Z_0_, eq 7, were characterized with bulk samples as described in the Methods section. For our STNO sample, we obtained at room temperature: *k* = 8.17 W m^–1^ K^–1^, *C* = 510 J kg^–1^ K^–1^, and ρ *=* 5110 kg m^–3^.

**Figure 2:**
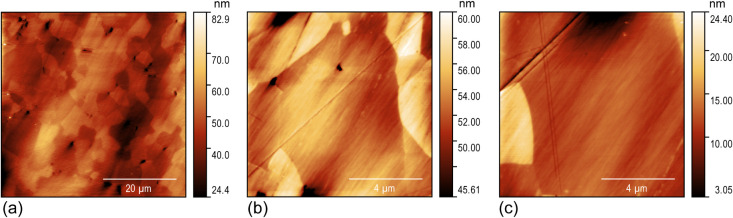
High-resolution topographic AFM images of the polished surface of the STNO ceramic sample. (a) 50 μm × 50 μm, (b) 10 μm × 10 μm, and (c) 10 μm × 10 μm.

The ceramic samples were fractured, and the fractured surfaces were polished (see the Methods for the description of the polishing procedure) to prepare them for the scanning probe microscopy (SPM) experiments. High-resolution atomic force microscopy (AFM) topographic images of the sample surface obtained in the tapping mode with sharp Si AFM probes (a nominal tip apex radius below 10 nm) are displayed in Figure 2. An SEM image of the polished surface is shown in Figure S1b. The root-mean-square (RMS) of the surface roughness with the account of waviness (waviness was defined as height variations with a lateral length scale exceeding 500 nm) was determined from the AFM image analysis to be about 0.20 nm, that is about half-unit-cell of the STO lattice.

#### 3.1.2 Probing Setup and Microheater Structure

**Figure 3:**
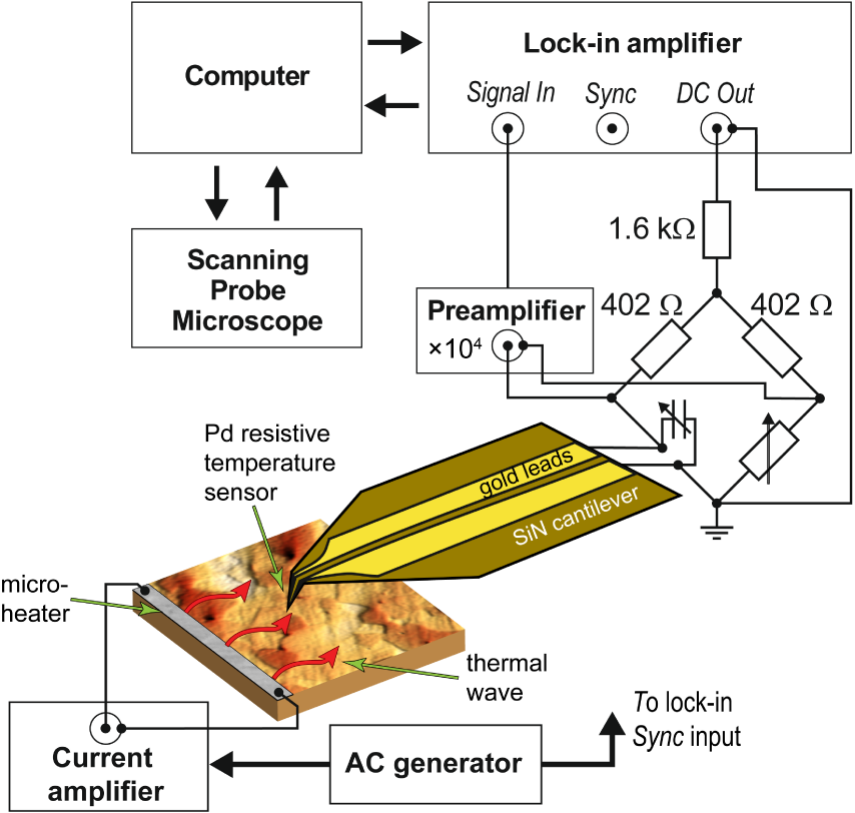
Measurement setup with the resistive SThM probe and block diagram of the signal detection circuit.

**Figure 4:**
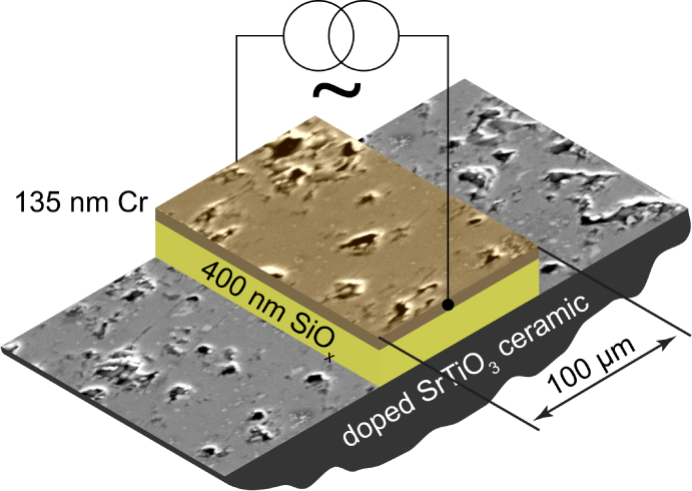
Schematics showing the structure of the microheater on the ceramic surface. The microheater is drawn as an overlay over an SEM image of the sample surface.

A schematic of the measurement setup is displayed in Figure 3. Thermal waves were created with the help of resistive microheaters fabricated on the sample surface using optical lithography and a liftoff process, as described in the Methods section. The cross-section of the double-layer structure of the microheaters is shown in Figure 4. The 400 nm layer of SiO_*x*_ and 135 nm of Cr were deposited on the ceramic with the e-beam evaporation. The SiO_*x*_ layer is electrically insulating to prevent the leakage of the current supplied to the heater into the semiconducting ceramic sample. The microheater width is 100 μm, and its length is about 2 mm. An AC current of a 100 Hz frequency was passed through the Cr layer of one of the microheaters, which resulted in the generation of thermal waves with the frequency of 200 Hz propagating in STNO along the normal to the microheater strip. The AC current amplitude was limited to 75 mA; larger currents and, hence, average heater temperature affected the stability of the STWM measurements. The scanning probe measurements were performed in the vicinity of one of the microheaters at a distance shorter than 25 μm from its edge. With a typical grain size in our ceramic sample in a range of 3–10 μm, such sample and test structure design provided a large “library” of grain boundaries for probing.

For localized measurements of the thermal wave amplitude and phase, commercially available thermoresistive SThM probes (Kelvin Nanotechnology KNT-SThM-2an, further denoted as “KNT”) were employed. The probes are equipped with Pd resistance temperature sensors fabricated on the probe tip. During measurement, the probe is in contact with the sample surface, and the sensor resistance is a function of the local sample temperature at the probe-sample contact. The probe is a part of a Wheatstone bridge circuit, as shown in the schematic in Figure 3. A small DC current (0.25 mA) is passed through a probe to detect changes in the electrical resistance of the Pd temperature sensor. The voltage from the output of the Wheatstone bridge is amplified with a precision low-noise preamplifier (homemade) and a lock-in amplifier (Zurich Instruments HF2LI). All measurements and imaging were performed in a vacuum at a pressure below 2 × 10^–3^ Torr so that the heat transfer by air can be neglected.

The maps of thermal wave amplitudes were acquired in the “jumping” mode SThM, which is described in detail elsewhere.^37^ In this mode, the measurements are performed with the probe in contact with a sample. However, the probe is lifted above the sample surface to be moved between pixels. A typical map was 100 px ×100 px. The jumping mode helps to reduce probe wear and contamination due to mechanical contact with the sample compared with the dragging motion of the probe between pixels in the conventional contact mode. The probe remained in contact with the sample surface for more than 1 s at each pixel of a map. This time is sufficient for the probe to relax (transition) into a steady state after the probe comes into contact with the sample. The relaxation could be controlled with the help of the AFM “Deflection” signal as well as steady component of the probe electrical resistance. After relaxation, 100 measurement readings were taken at each pixel; each pixel value in the maps is an arithmetic average of 100 readings. The scanning direction was at about 45° with respect to the heater strip.

### 3.2 Approximate Relations Between *R*_gb_ and the STWM Probe Response

Next, with the knowledge of the material properties, we can develop a simple analytical model as the first-order approximation for quantitative interpretation of the STWM measurements in our setup. In the model, we take advantage of the local character of the STWM probing. For that, first, we make an estimation of the mean free path (mfp) of phonons in our sample with the use of the expression provided, e.g., by Berman:^38^ mfp = 3*k*/*vCρ* ≈ 1.6 nm with the room-temperature sound velocity for STO of *v* = 5650 m/s.^39^ Such a phonon mfp is small compared to any characteristic size in our measurement setup. Hence, the heat transfer in the sample can be treated as diffusive, justifying the use of the Fourier law. Additionally, the suppression of *k* near the grain boundary, as observed in ref (7), can be ignored since it takes place at a distance of a few phonon mfp from the grain boundary.

At our measurement frequency *f* = 200 Hz, *l* = 50 μm and |Z_0_| = *l/k* = 6.1 × 10^–6^ K m^2^ W^–1^. As seen, |Z_0_| ≫ *R*_gb_≫ for values of *R*_gb_< 5 × 10^–8^ K m^2^ W^–1^, and to derive a simple relation for the determination of *R*_gb_ from local measurements (still working in the plane-wave approximation), we can apply the Taylor series expansion to the right-hand side of the eq 11 for the wave amplitude and leave only terms linear in *R*_gb_:
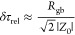
13

Hence,
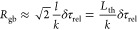
14

From eq 8, we can see that in our measurement, the “reflection” of thermal waves from grain boundaries is weak with |Γ| < 0.1 for boundaries with *R*_gb_ < 5 × 10^–8^ K m^2^ W^–1^.

In practice, the measurement points are located not in the immediate vicinity of the boundary as assumed in the equations above, but at some distances away from it, as schematically shown in Figure 1a. Such measurement will include the wave amplitude change across the corresponding thickness of the uniform material. In this case, the thermal resistance of the boundary can be found from the measured temperature amplitudes as
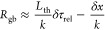
15where *δx* is the distance between measurement points. The last term on the right-hand side of eq 15 can be derived by linearizing the factor  in eq 4 and using the result in eq 10.

In turn, for the phase change δφ = φ_2_ – φ_1_, we have from eq 12:
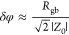
16

Hence,
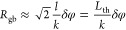
17

With measurement points at a distance from the boundary, by analogy with eq 15:
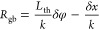
18

The plane wave approximation is illustrative, but it is too coarse to describe thermal waves in the geometry of our setup, which is closer to cylindrical. Therefore, we further consider a cylindrical wave model. In this model, we present a sample as a homogeneous semi-infinite medium and a grain boundary as a discontinuity along an infinite circular cylindrical surface surrounding the heater strip, as shown in Figure 1b. The axis of the cylindrical surface is in the sample surface. The heater strip is replaced by a linear uniform heat source at the axis of the cylindrical surface (shown as a small red circle in Figure 1b). The radial distance *r*_b_ between the linear heat source and the boundary is initially unknown and can be treated as a fitting parameter. The relations for determining the grain boundary resistance in the cylindrical geometry are derived in Section S2. Here, we write down only the result:
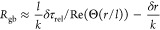
19and
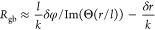
20where *r* is the radial coordinate of the cylindrical coordinate system with the axis at the linear heat source, and *δr* is the radial distance between measurement points.

**Figure 5:**
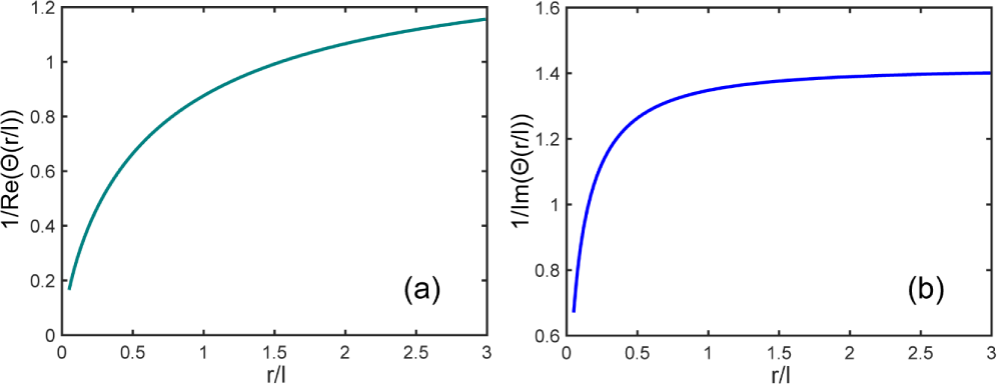
Plots of functions (a) 1/Re(Θ(*r*/*l*)) and (b) 1/Im(Θ(*r*/*l*)).

Plots of the functions 1/Re(Θ(*r*/*l*)) and 1/Im(Θ(*r*/*l*)) (defined in eq S5) are shown in Figure 5. Both the functions have  as an asymptote at *r* → ∞, which corresponds to the transition to plane waves (compare with eqs 15 and 18). As can be seen, their values can be approximated by unity at *r/l* > 0.2. This means that for estimations of *R*_gb_, values of the functions can be set equal to unity without accurate measurement of *r*_b_.

For a more accurate determination of *R*_gb_, including distance dependence of the coefficient at *l/k* in eqs 19 and 20, it is possible to perform integration over the heater strip width to account for the distributed nature of the heat source, as is done in ref (28). However, numerical modeling is better suited for samples with small grain sizes when an analytical model cannot be developed to cover a reasonable range of samples. In the next section, we discuss a basic numerical model for an isolated semispherical grain embedded into a matrix of the same material and introduce distance-dependent correction coefficients, which can be applied to obtain more accurate results.

### 3.3 Numerical Modeling of Signals Across Grain Boundaries

**Figure 6:**
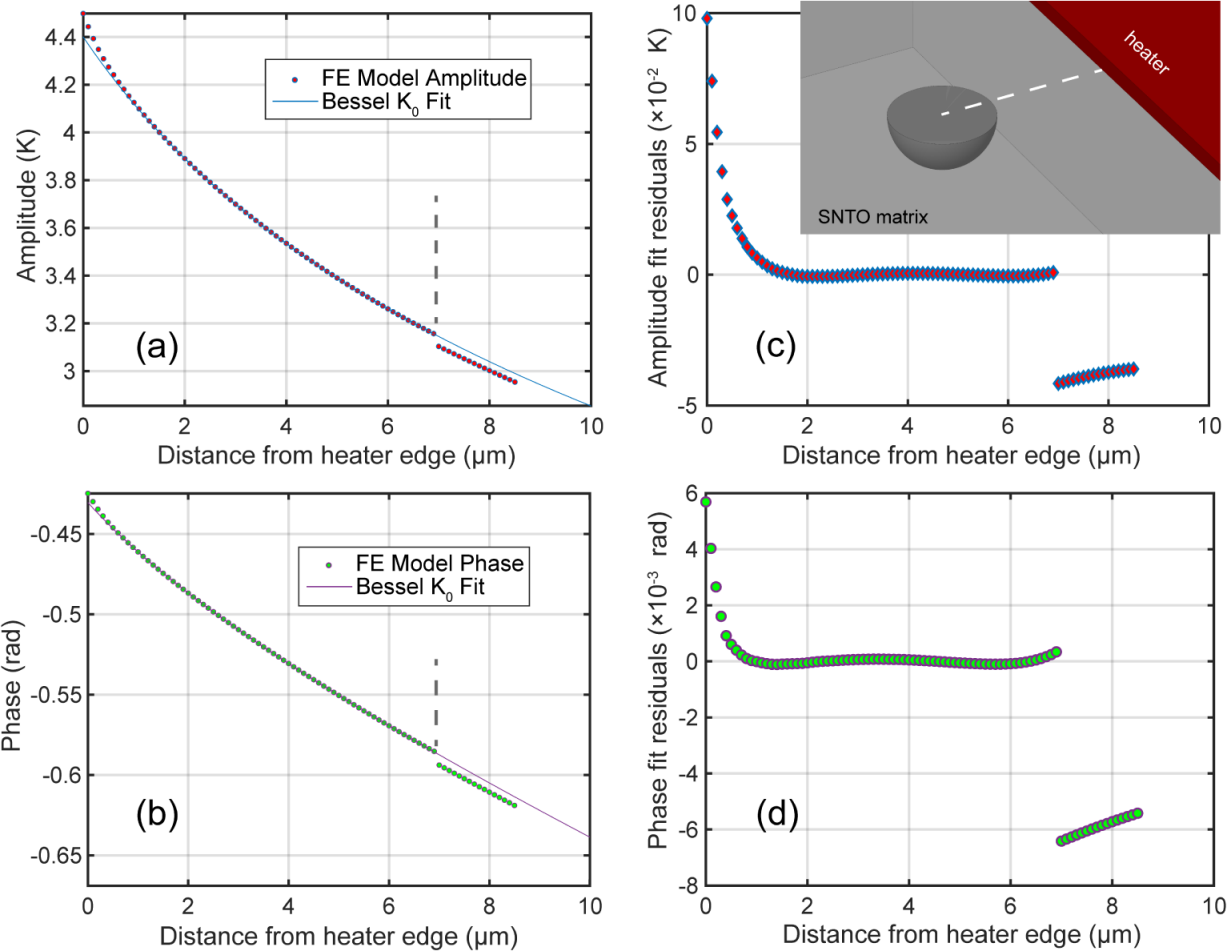
Typical results of the FE modeling. The layout of the FE model with the microheater and a semispherical inclusion representing a grain is shown as the inset in panel (c). (a) Amplitude and (b) phase as functions of the distance from the heater strip edge. Symbols are FE-calculated values, and solid lines are fits of eqs 21 and 22, respectively, to the calculated values outside of the inclusion. The dashed lines indicated the position of the grain (inclusion) boundary. (c) and (d) are fit residuals for the amplitude and phase, respectively. The heater-boundary distance in the FE model is *d*_hb_ = 7 μm, and *R*_gb_ = 5 × 10^–8^ K m^2^ W^–1^. The values for fitting parameters are for amplitude – *d*_0_ = −3.89 μm, *l* = 102.7 μm, and τ_0_ = 1.27 K, for phase – *d*_0_ = −1.56 μm, *l* = 56.5 μm, and φ_0_ = −0.22 rad.

The thermal wave generation in our experimental setup was numerically simulated with a finite-elements (FE) model. Examples of the simulation results are displayed in Figure 6. In the model, we introduced a heater strip with a vertical structure analogous to that used in the experiments. A partial layout of the model is shown in the inset in Figure 6c. The grains were modeled as semispherical inclusions of the same material as the matrix separated from the matrix by a boundary with a (varying) thermal resistance *R*_gb_. The grain radius was set to 4 μm, with some calculations made with grain radii of 2 and 5 μm to investigate the grain size effect, which turned out to be negligible. The material parameters were equal to the experimental ones (see Section S3 for details on the FE model and more simulation results).

The temperature oscillation amplitude and phase obtained in the FE model were extracted along the line indicated in the mode layout in the inset in Figure 6c that runs on the ceramic surface along the normal to the edge of the heater and through the inclusion center. For fitting the extracted amplitude and phase as functions of the distance from the heater edge, *d*, the following functional forms were employed (compare with eq S1 with *p* = 0):
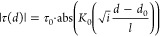
21for amplitude, and
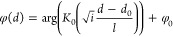
22for phase, with *d*_0_, *l*, τ_0_, and φ_0_ as fitting parameters. Here, *K*_0_(···) is the zeroth-order modified Bessel functions of the second kind. The points inside the inclusions were excluded from the fitting.

Figure 6a,b displays FE-calculated amplitude and phase (dots) as functions of the distance from the heater strip edge as well as corresponding fits of eqs 21 and 22 to the calculated data for the matrix part (solid lines). The heater-boundary distance in the model used for the plots is *d*_hb_ = 7 μm, and *R*_gb_ = 5 × 10^–8^ K m^2^ W^–1^. Figure 6c,d shows respective fit residuals. For the latter plots, the functional fits served as backgrounds, which are subtracted from the numerical data of Figure 6a,b. The plots in Figure 6c,d reveal the amplitude and phase jumps at the grain boundary, the effects of wave “reflection” (heat accumulation) at the boundary, as well as the effects of the proximity to the heater strip near the strip. In the vicinity of the heater edge, the plots of residuals show noticeable deviations from values obtained in the cylindrical geometry model: for amplitude up to a distance of about 1.5 μm from the heater edge and for phase up to a distance about three times smaller. The increase of the residual values near the grain boundary on the heater side from the boundary is apparently due to the heat accumulation near the boundary. As can be seen, the latter effect is weak in comparison with the jumps across the boundary. This was the case for *R*_gb_ = 5 × 10^–8^ K m^2^ W^–1^ as well as for other values of *R*_gb_ used in the simulations. Figure S3 displays similar plots for the heater-boundary distance of 17 μm and *R*_gb_ = 5 × 10^–9^ K m^2^ W^–1^. The plots show clearly that cylindrical geometry is a good approximation at larger distances from the heater.

However, it was observed that the thermal penetration depth *l* in the fits is different from that calculated based on the parameters of the sample material in the FE model. Namely, *l* in the amplitude fits varied systematically between 51 μm and about 250 μm for different heater-boundary distances with *l* = 50 μm when calculated with eq 5. The discrepancy is larger for larger *R*_gb_ and smaller *d*_hb_ (sharply dropping between *d*_hb_ = 10 μm and *d*_hb_ = 20 μm). The variation for phase was smaller—*l* in the fits varied between 50 and 100 μm—with the same trend vs boundary resistance and distance but with *l* < 60 μm already for *d*_hb_ > 6 μm. The value of the parameter *d*_0_ corresponds to the position of the substitute linear heat source, and it can be negative as well as positive for both amplitude and phase (for negative values, the heat source is under the heater strip), with |*d*_0_| < 10 μm and larger for larger heater-grain distances.

With such variability, despite the excellent fitting quality, we adapted a different approach to quantify *R*_gb_ from STWM maps. Namely, we introduced coefficients *h*_*τ*_ and *h*_*φ*_ preserving the functional forms of eqs 19 and 20:
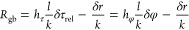
23

**Figure 7:**
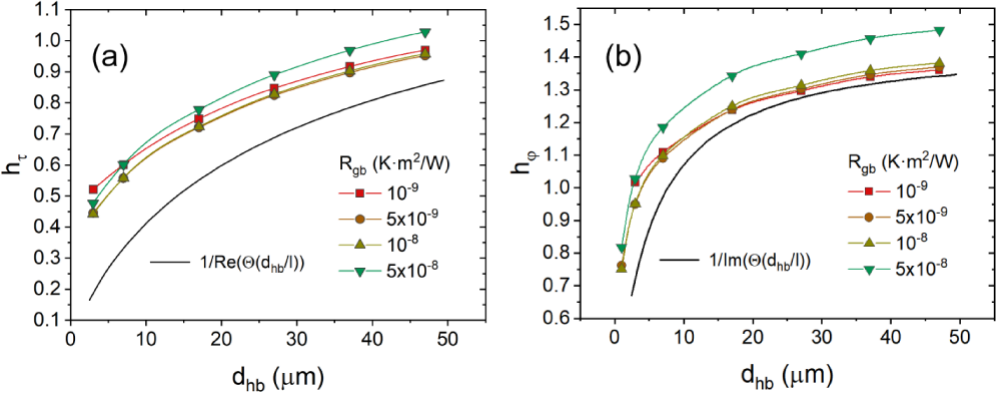
Solid lines with symbols: Plots of numerically calculated coefficients (a) *h*_τ_ and (b) *h*_φ_ defined by eq 23 for an inclusion radius of 3 μm. Different lines are data calculated with different values of *R*_gb_, as shown in the panel legends. Plain solid lines are plots of functions (a) 1/Re(Θ(*r*/*l*)) and (b) 1/Im(Θ(*r*/*l*)) (same as in Figure 5) with *r* = *d*_hb_ and *l* = 50 μm.

The coefficients *h*_*τ*_ and *h*_*φ*_ are determined with the use of eq 23 with *R*_gb_ values set in the FE models, *δr* = 0, and calculated jumps *δτ*_rel_ and *δ*_*φ*_. Figure 7 displays plots of *h*_*τ*_ and *h*_*φ*_ versus heater-grain boundary distance, *d*_hb_, obtained with the FE models for four values of *R*_gb_. As seen, the relative difference between the coefficient *h*_*τ,φ*_ and functions 1/Re(Θ(*r*/*l*)) and 1/Im(Θ(*d*_hb_/*l*)) decreases with increasing distance from the heater edge. The difference is larger for the large *R*_gb_ = 5 × 10^–8^ K m^2^ W^–1^, apparently, because the condition *R*_gb_*k/l* ≪ 1 is weaker fulfilled for this *R*_gb_ value. It is also clear that the function 1/Im(Θ(*d*_hb_/*l*)) can be used in place of *h*_*τ*_ for order-of-magnitude estimates with an accuracy of about 50% or better for *d*_hb_ > 0.1*l.* In turn, the function 1/Im(Θ(*d*_hb_/*l*)) better approximates values of; the approximation is nearly ideal for phase at larger *d*_hb_ and smaller *R*_gb_.

### 3.4 Experimental Results

Due to limitations of our STWM system, we cannot obtain amplitude and phase maps simultaneously. Therefore, here we focused on amplitude maps; phase maps gave close results. Multiple maps were obtained across the sample near the microheater strips. Decay of the thermal wave amplitude strongly dominates the contrast in the raw-signal as-acquired maps. To reveal the signal difference between grains, the raw-signal maps were leveled by the mean plane subtraction. Only a few maps showed a clear contrast that could be associated with the grain boundary thermal resistance.

**Figure 8:**
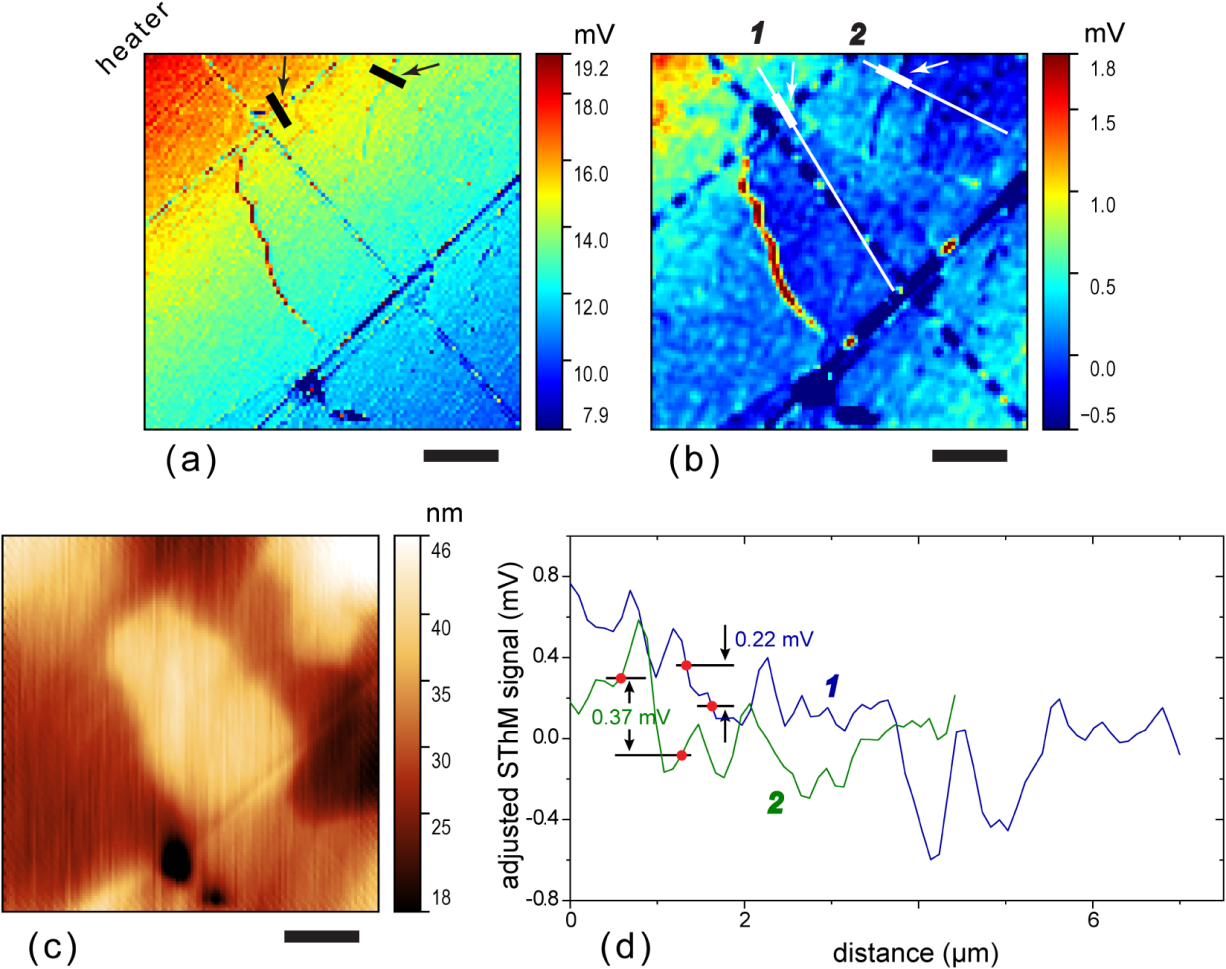
STWM maps with grain boundaries located about 9 μm from the heater. (a) Raw, unprocessed map of the probe signal amplitude at the input of the lock-in amplifier; locations that were used for quantification of *R*_gb_ are marked with short, thick black lines. (b) The map in (a) after leveling and application of a Gaussian filter with a 2 px-wide window; locations that were used for quantification of *R*_gb_ are marked with short, thick line segments on top of thinner lines. (c) A topographic map of the same area as in (a), which was acquired in the tapping mode with the same SThM probe. (d) Cross-sectional profiles along lines 1 and 2 in panel (b). The end points of the thick lines in (a) and (b) correspond to pixels where the signals for quantification of the *R*_gb_ was used as illustrated with dots on the profile lines in (d). Scale bars in (a)–(c) are 2 μm, and the size of the scanned area is 10 μm × 10 μm.

Figure 8 illustrates one example. The map in the figure was taken with about 12 μm between the heater edge and the middle of the scanned area of 10 μm × 10 μm in size. Figure 8a shows the raw-signal map of the probe response as voltage amplitude at the lock-in amplifier input. The corresponding map after leveling is displayed in Figure 8b. The leveled map was additionally smoothed by applying a Gaussian filter with a 2 px-wide window. After the STWM map was acquired, the topographic map in Figure 8c was obtained in the standard tapping (intermittent contact) mode using the same SThM probe. The areas with the reduced response amplitude in Figure 8b correspond to individual grains, as becomes clear after comparing the maps in Figure 8b,c.

Positions of the boundaries were identified in the leveled STWM map, Figure 8b, and, after that, transferred into the raw-signal map, Figure 8a. Due to a strong noise in the images, the values selected for determination of the thermal boundary resistance were obtained by averaging values at pixels close to the boundaries on both sides, omitting features due to the apparent topographic crosstalk (predetermined by the static topography of the surface). Then, pairs of pixels with the values closest to the average ones were selected. The selected pixels are at the ends of the short, thick-line segments indicated with arrows in the maps. They are also shown in Figure 8d on the cross-sectional profiles taken along the lines indicated in the leveled map in Figure 8b. The values of |τ| and corresponding in-between-pixels distance *δr* to use in the calculation were read from the raw-data map, Figure 8a. The boundary-heater distances for both boundaries in the maps are about 9 μm. From plots in Figure 7a, we determine h_τ_ ≈ 0.6 for this distance and find *R*_gb_ values with eq 23: *R*_gb_ = 2.2 × 10^–8^ K m^2^ W^–1^ for boundary 1 and *R*_gb_ = 1.6 × 10^–8^ K m^2^ W^–1^ for boundary 2.

**Figure 9:**
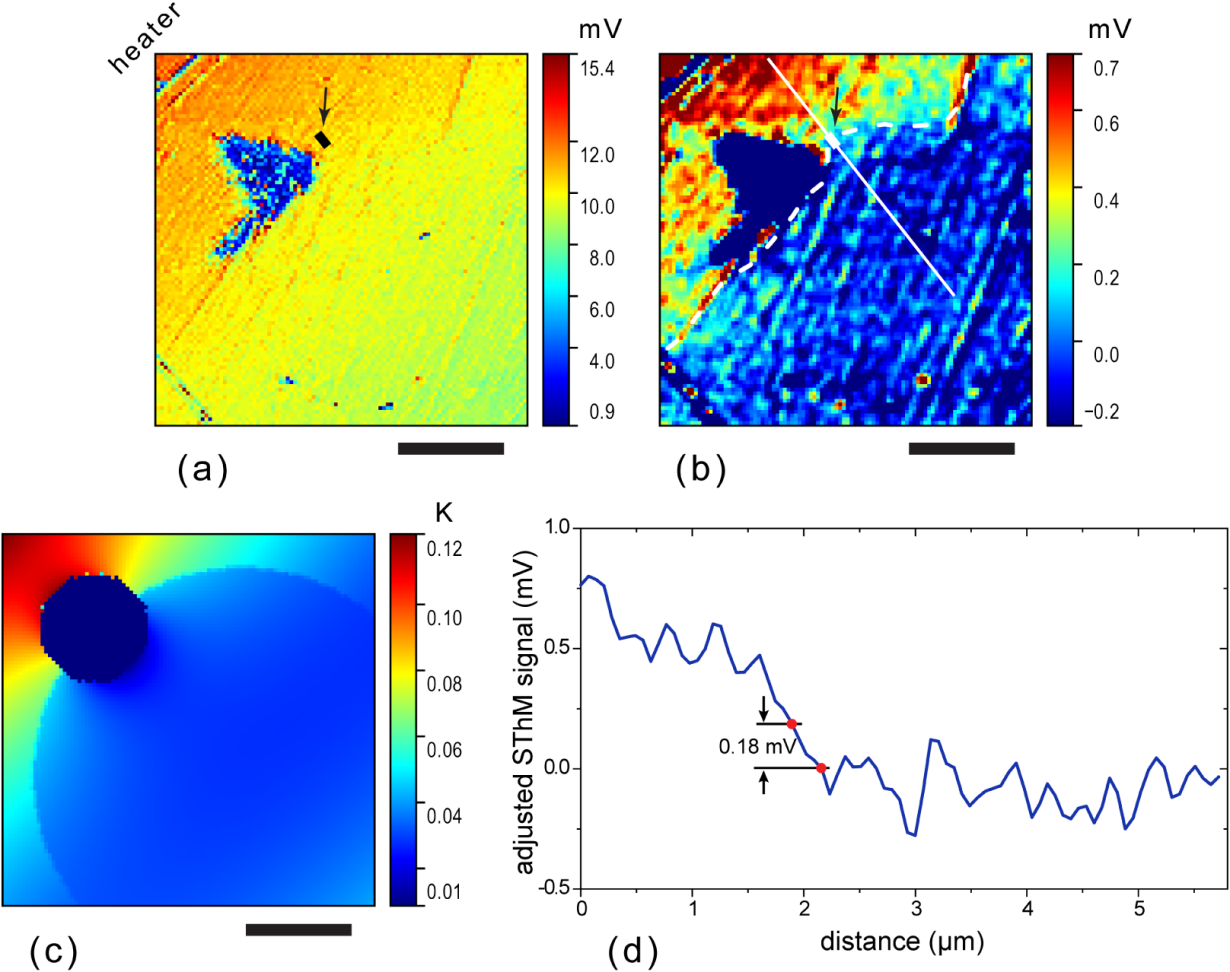
STWM maps with a grain boundary located 17 μm from the heater. (a) Raw, unprocessed map of the probe signal amplitude at the input of the lock-in amplifier; the location that was used for quantification of *R*_gb_ is marked with the short line segment indicated with an arrow. (b) The map in (a) after leveling and application of a Gaussian filter with a 2 px window; locations that were used for quantification of *R*_gb_ is marked with a short, thick line segment on top of a thinner line. The dashed line indicates the grain boundary. (c) A map simulated with an FE model imitating the sample in (b). The map is leveled; the FE model layout and the raw calculated map are provided, Figures S5 and S6b, respectively. (d) Cross-sectional profiles along the straight line in panel (b). The end points of the thick line segments in (a) and (b) correspond to pixels where the signals for quantification of the *R*_gb_ was used as illustrated with dots on the profile in (d). Scale bars in (a)–(c) are 2 μm, and the size of the scanned area is 7 μm × 7 μm.

Another example of a boundary showing a change in the thermal wave amplitude is shown in Figure 9a,b. The maps are 7 μm × 7 μm in size and the distance between the heater edge and the middle of the scanned area is about 18 μm. The leveled map in Figure 9b clearly shows the wave amplitude jump across the grain boundary. The corresponding topographic image is provided in Figure S6a. This grain boundary passes through a pore. The presence of the pore required an additional correction of the values obtained with eq 23, and we have numerically modeled the thermal wave with the boundary passing a pore of a similar size. The FE model of a grain with a pore is shown in Figure S5, and the as-calculated map is in Figure S6b. Figure 9c shows a leveled simulated map for comparison with the experimental one in Figure 9b. There is a clear resemblance between experimental and simulated maps, which supports the interpretation of the contrast seen in the experimental image as a change of the thermal wave amplitude due to the thermal resistance of the grain boundary. We have estimated the grain boundary thermal resistance similarly to that in Figure 8. The pixels selected for the estimation correspond to the ends of the thick line segments indicated with arrows in the raw-signal and leveled maps, Figure 9a,b. The pixels are also indicated in Figure 9d, which displays the cross-sectional profile along the line denoted in the leveled map in Figure 9b. The boundary-heater distance for this boundary is about 17 μm. From plots in Figure 7a, we find *h*_*τ*_ ≈ 0.75 for this distance. The FE-calculated correction factor accounting for the pore is *h*_pore_ ≈ 0.7; hence, *h*_*τ*_ → *h*_*τ*_*h*_pore_ ≈ 0.75 × 0.7 ≈ 0.5, and we find with eq 23: *R*_gb_ = 3.3 × 10^–8^ K m^2^ W^–1^.

### 3.5 Discussion and Outlook

The values obtained above can be compared with the data of ref,^26^ where measurements of grain boundary thermal resistance were performed on CeO_2_ ceramics. In ref (26), the grain boundary resistance covers a range from 2 × 10^–9^ K m^2^ W^–1^ to about 2 × 10^–8^ K m^2^ W^–1^. The estimated *R*_gb_ values found in our STNO ceramics suggest that we were able to detect the most resistive boundaries in our sample, which are relatively rare, with the thermal resistance of most, “irresponsive” boundaries in our sample being below 10^–8^ K m^2^ W^–1^.

It can be assumed that the responses from boundaries with lower resistances are buried in the large noise in the images. The noise RMS in the maps in Figures 8a and 9a is 200–250 μV. To determine the source of this noise, we measured the noise of our measurement circuit at the input of the lock-in amplifier and calculated its contribution to the noise of the mapped signal. The resulting value is 9.8 μV RMS in the maps. This value can be further represented as the measurement system sensitivity or threshold detectable *R*_gb_, which yields *R*_gb_ 3.4 × 10^–9^ K m^2^ W^–1^. (For measurement and calculation details see Section S4.) The value can be further reduced, for instance, by averaging over a larger number of data points at one pixel, increasing the probe current used to measure probe resistance, which increases the signal, or by more advanced signal processing. However, despite the quite heavy-averaging algorithm used to determine *R*_gb_ in the experiments, the experimental *R*_gb_ values are significantly larger than 3.4 × 10^–9^ K m^2^ W^–1^ deduced based on the noise of the measurement electronics. In turn, the noise RMS of the signals in the maps in Figures 8a and 9a is about 20–25 times larger than the contribution of the noise of the electronics.

The origin of the large noise becomes clear after a comparison of the STWM images and images of sample topography in Figure 2, where one can see a dense, hair-like, texture of scratches left from polishing on the sample surface. A similar texture is visible through noise in the raw-signal STWM maps in Figures 8a and 9a. Furthermore, the noise/signal ratio in the STWM images is approximately constant and independent of the signal level across maps acquired at different distances from the microheater. The latter indicates that the noise in the STWM maps is caused by the variation of the probe-sample contact thermal resistance due to the residual surface roughness after polishing. It is worth noting that the depth of individual scratches is small—0.3 to 0.9 nm—that is, about 1–2 unit cells of the STO lattice. Still, the SThM images show a relatively large level of pixel-to-pixel noise, which prohibits accurate localization of a grain boundary and measurements of the signal jumps across the boundary. Apparently, the contact-resistance noise associated with surface roughness cannot be averaged out by increasing the integration time or sample temperature amplitude.

**Figure 10:**
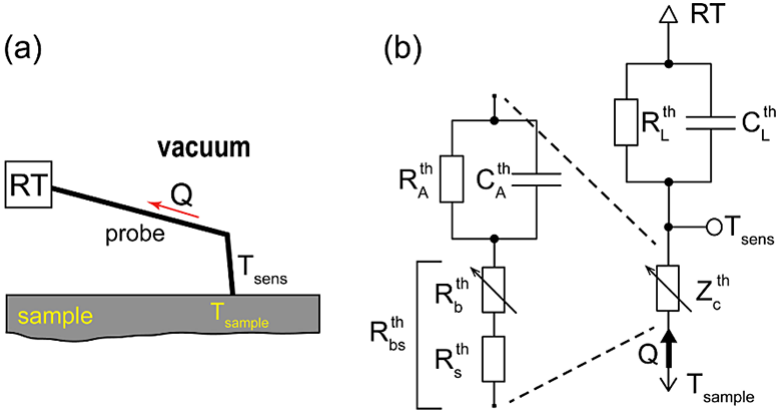
(a) A schematic of the probe in contact with a sample and (b) equivalent lumped-elements thermal circuit of the probe-sample system. See the text for the meaning of the elements. *Q* is the heat flux from the sample through the probe to the environment, *RT*. *RT* stands for room temperature.

To elucidate the source of the large roughness-related noise, we analyzed the probe-sample system performance with a focus on variation of the probe-sample contact thermal resistance. Figure 10 shows a schematic of the probe in contact with a sample and the equivalent lumped-elements thermal circuit of the probe-sample system. The output of the circuit, *T*_sens_, corresponds to the temperature of the thermoresistive sensor. The sensor is thermally connected to a sample with a temperature *T*_sample_ through the probe tip apex represented by the  pair, probe-sample boundary thermal resistance, , and the spreading thermal resistance of the sample at the contact, , . The part of the probe between the thermosensitive sensor and the probe attachment point, which we call “lever”, is represented by the pair . Capacitors  and  account for the thermal capacitances of the relatively massive probe tip and lever, respectively. The effect of these capacitances increases with increasing frequency of sample temperature oscillations, which leads to a drop in the probe sensitivity above a cutoff frequency. The cutoff frequency of the KNT probes is about 10 kHz (see Section S5, for FE modeling of the frequency response of the probe).

From the schematic in Figure 10b, it is clear that the probe functions analogously to a resistive voltage divider, where the probe sensor temperature (output “voltage” of the divider) is a fraction of the sample temperature (input “voltage” of the divider). The probe response is proportional to the ratio 1/(1 + *s*), where , and  and are complex thermal impedances of the “contact” and “lever” parts of the probe, respectively. For the KNT probes used in this work, ,^37^ that is, , and, hence, |*s*| ≫ 1, which makes the probe less sensitive to sample temperature and simultaneously very sensitive to variations of the probe-sample boundary resistance. This applies similarly to the amplitude and phase of the signal (see FE-simulated probe response amplitude and phase as functions of  in Figure S8c).

To be more specific, based on our own results with ceramic samples reported in ref (37), we can assume that at low frequencies,  K W^–1^, and  K W^–1^ for the average probe-sample thermal resistance in our experiments. Therefore, *s* ∼ 10 ≫ 1. The relative value of the noise (the noise-to-signal ratio) in the images in Figures 8a and 9a is 1–3%. In the linear approximation, the relative variation of the probe response is s/(1+*s*) times the relative variation of *s*. Therefore, with *s* ≫ 1, the noise of 1–3% of the full signal, as in the maps in Figures 8a and 9a, is caused by only 1–3% variations of the probe-sample contact resistance. Hence, for sensitivity to be limited by the electronics, the contact resistance stability should be better than 10^–3^, which is difficult to ensure with ordinary surface polishing. As a possible solution, the sensitivity to  can be reduced and potentially eliminated by an increase of  so that . Ideally,  should be increased up to infinity, meaning the full thermal isolation of the temperature sensor of the probe from the environment. However, currently, there is no feasible probe design to fulfill such a condition since the probe should have a structure ensuring the probe-sample contact without compromising the mechanical integrity of the probe. Alternatively, the  and  can be reduced by increasing the probe-sample contact size at the cost of decreased spatial resolution.

Along these lines, it is of interest to compare this situation with that in the contactless optical methods based on modulated photothermal reflectance. In Time-Domain Thermoreflectance (TDTR) and Frequency-Domian Thermoreflectance (FDTR), a metal, light-reflecting film is deposited on top of the sample surface and is used as the thermal sensor, whose temperature is interrogated by detecting light reflection variations from it. Therefore, in the optical, contactless methods, the “lever” is absent, and the sensor is isolated from the environment. Furthermore, the analogs of  and  are very small, and is nearly constant across a sample. This allows using temperature modulation frequencies in the MHz range, with the signal phase being hardly affected by the sample topography.^27^ The comparison is not full if we do not include the amount of the probed sample material (pixel size) into consideration. The resolution of the STWM is ∼100 nm in our experiments, which can be deduced from the topographic images similar to that shown in Figure 8c. This is nearly equal to the probe-sample contact diameter. In turn, in the photothermal reflectance techniques, the minimal laser spot size is 1 μm, that is, 10 times larger. A hypothetical increase of the STWM probe-sample contact size to 1 μm will result in a 100-fold reduction of  with a comparable reduction of the probe sensitivity to the  variations. Hence, at the 1 μm probe-sample contact size, one may expect a comparable performance of STWM and photothermal reflectance-based techniques.

SThM probes with sensors thermally isolated from the environment are currently unavailable. Probes with a controllable probe-sample contact size are unavailable as well. However, some methods to overcome the effects of the  variations in the temperature mapping with scanning probes were proposed in the literature and can be applied in future work. The one most promising for implementation in STWM was suggested and tested by Menges and co-workers.^40,41^ Menges et al.^41^ proposed a measurement scheme that allows simultaneous measurements of sample temperature and probe-sample contact thermal resistance in one probing run to determine the true sample temperature free of the influence of the probe-sample contact resistance. In their measurement procedure, probe-sample contact resistance is accounted for by passing a DC current through a thermoresistive probe. Heating the probe with a DC current makes it active, similar to the active-mode SThM, where the probing is used primarily to map .^42^ In our thermal-wave measurements, we have three unknowns to determine: static and oscillating sample temperatures as well as probe-sample contact resistance. The method of Menges et al. can be modified so that the three unknowns can be found by passing an AC current through the probe at a frequency different from the frequency of a thermal wave. The probe signal should be detected at DC and both the AC frequencies. The three signals can be used to account for the probe-sample contact thermal resistance and calculate the true sample temperature and amplitude of the thermal wave from the other two signals. Implementation of this approach is a subject of a future work.

## 4 Conclusions

To summarize, we have applied scanning thermal wave microscopy to measure the thermal resistance of grain boundaries in polycrystalline materials. The high spatial resolution of this scanning probe method allows it to be employed for finely grained materials. As a model material system, we studied a perovskite oxide ceramic material, Nb-substituted SrTiO_3_, with an average grain size of approximately 5 μm. In the method, we induce thermal waves within the sample using a microheater fabricated on its surface and map the resulting thermal wave field with a temperature-sensitive scanning probe. One of the key advantages of scanning probe microscopy is the ability to make highly localized point-by-point measurements, enabling the examination of thermal fields in the immediate vicinity of grain boundaries. This localized probing simplifies the data analysis. To quantify the thermal resistance of grain boundaries, we employed a simple, linearized, analytical model as a first-order approximation, assessing changes in thermal wave amplitude and phase across these boundaries. To refine our results, we utilized numerical simulations.

We have achieved a detectability limit for the grain boundary thermal resistance of approximately about 2 × 10^–8^ K m^2^ W^–1^, making the method directly applicable to chalcogenides-based thermoelectric materials, where typical grain boundary thermal resistances are about 10^–8^ K m^2^ W^–1^ on the order of magnitude. However, grain boundaries in oxides, where typical grain boundary thermal resistance is lower, demand higher detectability. A significant obstacle to achieving this higher detectability lies in the probe sensitivity to variations in probe-sample contact thermal resistance. These variations arise from the relatively low thermal resistance between the probe and its environment, combined with significant thermal resistance at the probe-sample contact. In the future, techniques to mitigate these effects can be applied. Nevertheless, considering the spatial resolution and the amount of material involved in detection, the sensitivity of our scanning probe method in its current embodiment is at least comparable to that of optical thermoreflectance methods, and the method opens up a door to characterization of thermal resistance at the level of single grain boundaries and domain walls in a spectrum of microstructured materials. Reduction of the grain size below the 5 μm used here will not affect the measurement principle. However, it should be kept in mind that the thermal wave field structure may be affected by densely spaced grain boundaries, which should be accounted for in a numerical model. The extent and intensity of this effect, however, are material-dependent and can be controlled by the thermal wave frequency.

## 5 Methods

### 5.1 Ceramic Sample Synthesis

The synthesis and processing of STNO ceramics are detailed in ref (36) and summarized here. A conventional solid-state route was used to prepare Nb-substituted SrTi_0.9_Nb_0.1_O_3_ powders from SrCO_3_ (Sigma-Aldrich, ≥ 99.9%), TiO_2_ (Sigma-Aldrich, 99.8%) and Nb_2_O_5_ (Aldrich, 99%). Before weighing, titanium and niobium oxides were annealed at 973 K for 2 h in air to remove the moisture and adsorbed CO_2_. The precursor powders were mixed in stoichiometric proportion and were annealed at 1173, 1373, 1473, and 1573 K for 5 h at each temperature with multiple intermediate regrindings. After final ball-milling with ethanol and drying, the disc-shaped samples were compacted using consecutive uniaxial and isostatic pressing. A two-step sintering approach to producing ceramics with ∼95% density, ρ included presintering in the air at 1973 K for 10 h, followed by a reduction in 10%H_2_-90%N_2_ atmosphere at 1773 K for 10 h. For the measurements of thermal diffusivity, *D*_th_, 1 mm-thick disc-shaped ceramics were prepared. For measurements of the specific heat capacity, *C*, the ceramics were converted to powder.

### 5.2 Measurements of Thermal Diffusivity and Specific Heat Capacity

The thermal diffusivity, *D*_th_, and specific heat capacity, *C*, of the ceramic sample were measured respectively with the use of a Netzsch LFA 457 Microflash system and Netzsch DSC 404 C differential scanning calorimeter (Netzsch, Germany) in flowing 10%H_2_-90%N_2_ mixture on stepwise cooling from 1273 to 373 K, followed by up to 1 h equilibration at each temperature. The data were extrapolated to 300 K (room temperature). The thermal conductivity, *k*, was calculated as *k* = *D*_th_*ρC*. For the STNO sample reported in this paper, we obtained at room temperature: *k* = 8.17 W m^–1^ K^–1^, *C =* 510 J kg^–1^ K^–1^, ρ = 5110 kg m^–3^.

### 5.3 Sample Surface Polishing

The ceramic composition was selected taking into account that any inclusions in the ceramics can interfere with surface polishing, causing roughening of the sample surface, which makes impossible meaningful SPM measurements as a result of a high density of deep scratches. For polishing, the ceramic pellets were placed in a mold and filled with epoxy glue EpoThin 2 (Buehler, Switzerland), followed by drying the glue in a low vacuum (at a pressure below 10^–1^ Torr) for 12 h. The resulting tablets were then polished using silicon carbide grinding papers and abrasive diamond paste, with particle sizes progressively decreasing down to 0.25 μm. Fine polishing was carried out with a 60 nm-size colloidal silica-based alkali solution (SF1 Polishing Suspension, Logitech, United Kingdom) for approximately 30 min to remove any mechanically strained regions from the sample surface that may have appeared after previous polishing steps. Subsequently, the sample surface was cleaned with acetone and isopropanol, followed by optical lithography steps to define electrodes on the surface.

### 5.4 Fabrication of Microheaters

The microheaters were fabricated with lithography and a liftoff process. The AZ ECI 3027 photoresist was spin-coated using the Sawatec SM-180/HP-250-HDMS unit (Sawatec, Switzerland) at a speed of 2000 rpm, with an acceleration time of 0.3 s and a duration of 30 s. The resist was prebaked by heating at a rate of 3 °C/min from room temperature and soaking at a temperature of 80 °C for 1 min. The SUSS MJB4 mask aligner (SUSS MicroTec, Germany) was utilized for aligning the mask and photoresist exposure. The resist was exposed to UV radiation for 100 s with an intensity of around 6 mW/cm^2^. Development of the resist was carried out using an AZ MIF 726 solution for 1 min resulting in the formation of openings in the photoresist layer in the form of 100 μm-wide stripes ready for the deposition of the microheater layers. For electrode sputtering, an Elato 350 vacuum unit (Izovac, Belarus) was employed. Layers of SiO_*x*_ and Cr, with thicknesses of 400 and 135 nm, respectively, were deposited onto the sample surface using electron beam evaporation. Following deposition, the liftoff was made by soaking the sample in DMSO (C_2_H_6_OS) at 60 °C for 10 min.

### 5.5 AFM Measurements

The SThM setup was assembled in-house and mounted on a scanning probe microscope NTEGRA Aura (NT-MDT, Russia). KNT-SThM-2an resistive probes (Kelvin Nanotechnology, UK, obtained from NanoAndMore, GmbH) with a nominal tip radius below 100 nm and a spring constant of 0.45 N/m were utilized for the STWM. The probe-sample contact force was set to 3 nN during all STWM experiments. The measurements were conducted under a vacuum of 2 × 10^–3^ Torr. To realize scanning thermal wave microscopy, heating for the generation of thermal waves was achieved by passing a 100 Hz AC current with an amplitude of 75 mA through a microheater on the sample surface, resulting in a total static surface heating of around 80 °C near the microheater edge. For that, the AC voltage from an auxiliary output of the lock-in amplifier (Zurich Instruments HF2LI, Switzerland) was applied to the microheater through a current amplifier (Accel Instruments TS-250, USA). A “jumping mode” SThM was employed to minimize the degradation of the resistive probe when interacting with surface topography.^37^ Complementary topographic images of the ceramic surface were acquired in the tapping mode at higher-order noncontact flexural resonances using the same SThM cantilevers as were used for corresponding STWM images. High-resolution AFM images were obtained in the tapping mode with sharp silicon cantilevers (Nanosensors PPP-NHCR, Switzerland) with a nominal tip apex radius below 10 nm. The images were analyzed with Gwyddion v. 2.61 software. Further details of the SThM setup can be found elsewhere.^37^

### 5.6 Finite-Element Modeling

The numerical modeling of the thermal wave propagation and of the KNT probe dynamic response was carried out with the use of COMSOL Multiphysics v.5.3a (COMSOL AB) finite elements analysis package as described in detail in Sections S3 and S5.

## Supporting Information Available

Supplementary SEM images of the sample surface, derivation of equations for the cylindrical geometry, description of the FE model for thermal wave in the vicinity of a grain, additional results of FE modeling, description of the characterization of the noise of electronics and estimation of measurements sensitivity, and FE modeling of the dynamic response of the KNT probe (PDF)
