# Supplementary material for: Nanoscale Imaging and Measurements of Grain Boundary Thermal Resistance in Ceramics with Scanning Thermal Wave Microscopy
Source: ACS Appl Mater Interfaces. 2024 Aug 5;16(32):42917–30. doi: 10.1021/acsami.4c08085 (PMC11331444; doi:10.1021/acsami.4c08085)
Supplement: Supplementary file 1 — am4c08085_si_001.pdf [file am4c08085_si_001.pdf]

## *Supporting Information*

# Nanoscale Imaging and Measurements of Grain Boundary Thermal Resistance in Ceramics with Scanning Thermal Wave Microscopy

Denis Alikin,[1] Maria J. Pereira,[1] Alexander Abramov,[2] Elena Pashnina,[2] Maria Chuvakova,[2] Nickolay V. Lavrik,[3] Wenjie Xie,[4,5] Anke Weidenkaff,[4,5] Andrei L. Kholkin,[1] Andrei Kovalevsky,[6] Alexander Tselev[1*]

[1]*Department of Physics & CICECO−Aveiro Institute of Materials, University of Aveiro, 3810-193 Aveiro, Portugal*

[2]*School of Natural Sciences and Mathematics, Ural Federal University, 620000 Ekaterinburg, Russia*

[3]*Center for Nanophase Materials Sciences, Oak Ridge National Laboratory, Oak Ridge, TN 37831, USA*

[4]*Materials and Resources, Department of Materials and Earth Sciences, Technical University of Darmstadt, 64287 Darmstadt, Germany*

[5]*Fraunhofer Research Institution for Materials Recycling and Resource Strategies IWKS, 63755 Alzenau, Germany*

[6]*Department of Materials and Ceramic Engineering & CICECO – Aveiro Institute of Materials, University of Aveiro, 3810-193 Aveiro, Portugal*

[*]Corresponding author, email: atselev@ua.pt

## S1. Supplementary SEM images

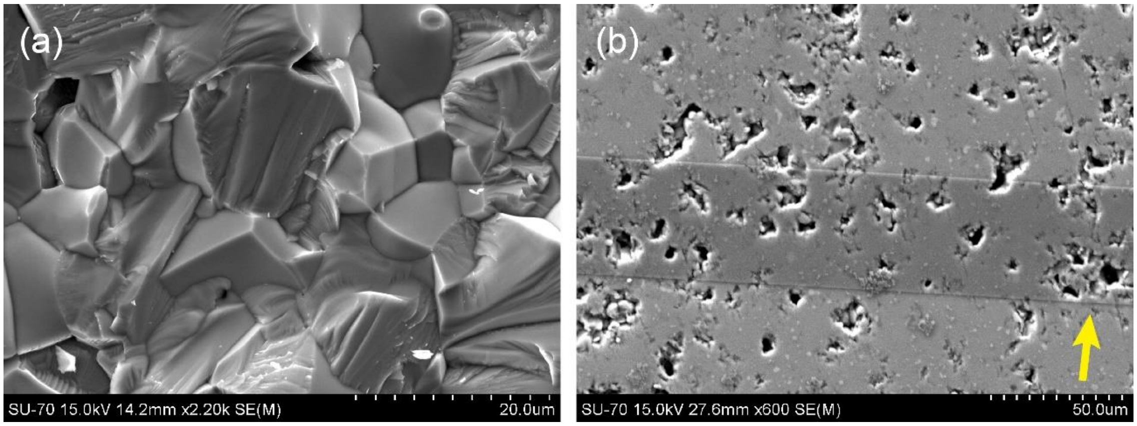


**Figure S1.** (a) Scanning Electron Microscopy (SEM) image of a fractured surface of a STNO ceramic sample. (b) SEM image of a polished surface. The darker horizontal stripe of a 50 μm width in the middle of the image is a microheater in one of its earlier versions. The arrow shows a site with pore agglomeration, which can lead to an "open circuit" in the heater stripe.

## S2. Derivation of Equations for the Cylindrical Geometry (eqs (19) and (20) of the main text)

The problem of periodically oscillating thermal field in the geometry of an infinite circular cylinder has a general analytical solution through modified Bessel functions with:[1]

$$\tau(r) = P \cdot I_0\left(\sqrt{i}\frac{r}{l}\right) + Q \cdot K_0\left(\sqrt{i}\frac{r}{l}\right). \tag{S1}$$

Here, $r$ is the radial coordinate of the cylindrical coordinate system, $I_0(\ldots)$ and $K_0(\ldots)$ are the zeroth-order modified Bessel functions of the first and second kind, respectively, $P$ and $Q$ are coefficients. Based on eq (S1), the length $l$ can be called *thermal penetration length*. In the cylindrical wave, the wavelength is not constant along the radius $r$, growing with $r$ and asymptotically approaching the value of the plane wave with $r \to \infty$. In the cylindrical wave, $\lambda_{\text{th}} = 0.6 \cdot 2\sqrt{2}\,\pi l$ at $r = 0.1l$, $\lambda_{\text{th}} \approx 0.9 \cdot 2\sqrt{2}\,\pi l$ at $r = 0.5l$, and $\lambda_{\text{th}} \approx 0.95 \cdot 2\sqrt{2}\,\pi l$ at $r = l$.

The infinite linear heat source in Figure 1b of the main text is at $r = 0$. In eq (S1), the first term diverges with $r \to \infty$, and the second terms goes to zero with $r \to \infty$. Therefore, in an unform media without discontinuities, $P = 0$. Considering the boundary as a weak "effective reflector", we take $P = 0$, which neglects heat wave accumulation (and increase of the wave amplitude) on the source side of the boundary in comparison with the amplitude change (jump) across the boundary. With this assumption, we have:

$$\tau_1 - \tau_2 = R_{\text{gb}} q \tag{S2}$$

with $q$ being the heat flux across the boundary:

$$q = -k\,\frac{\partial \tau}{\partial r} = \sqrt{i}\,\frac{k}{l}\,\frac{K_1\left(\sqrt{i}\,r/l\right)}{K_0\left(\sqrt{i}r/l\right)}\tau \qquad \text{(S3)}$$

where the derivative is taken in the vicinity of the boundary. In eq (S3), we used the equality $K_0'(z) = -K_1(z)$, where $K_1(z)$ is the first-order modified Bessel function of the second kind.[2] Due to the flux conservation across the boundary, from eqs (S2) and (S3) we obtain:

$$\frac{\tau_1 - \tau_2}{\tau_1} = R_{\mathrm{gb}}\frac{k}{l}\,\sqrt{i}\,\frac{K_1\left(\sqrt{i}\,r/l\right)}{K_0\left(\sqrt{i}r/l\right)} \qquad \text{(S4)}$$

For compactness, we introduce the function:

$$\Theta(r/l) = \sqrt{i}\,\frac{K_1\left(\sqrt{i}\,r/l\right)}{K_0\left(\sqrt{i}r/l\right)} \qquad \text{(S5)}$$

and re-write eq (S4) as:

$$\frac{\tau_2}{\tau_1} = 1 - R_{\mathrm{gb}}\frac{k}{l}\Theta(r/l) \qquad \text{(S6)}$$

For temperature oscillation amplitudes, we have:

$$\frac{|\tau_2|}{|\tau_1|} = \mathrm{abs}\left(1 - R_{\mathrm{gb}}\frac{k}{l}\Theta(r/l)\right), \qquad \text{(S7)}$$

and since $|\Theta(r)| \sim 1$, and $R_{\mathrm{gb}}/|Z_0| = R_{\mathrm{gb}}\,k/l \ll 1$:

$$\frac{|\tau_2|}{|\tau_1|} \approx 1 - R_{\mathrm{gb}}\frac{k}{l}\mathrm{Re}\big(\Theta(r/l)\big), \qquad \text{(S8)}$$

where we applied: $\sqrt{(1-a^2)+b^2} \approx 1 - a$ if $a, b \ll 1$. $\mathrm{Re}\big(\Theta(r/l)\big)$ is the real parts of the complex-valued function $\Theta(r/l)$. After a simple rearrangement in eq (S8) with $\delta\tau_{\mathrm{rel}}$ as defined in eq (10) in the main text, we obtain:

$$R_{\mathrm{gb}} \approx \frac{l}{k}\delta\tau_{\mathrm{rel}}/\mathrm{Re}(\Theta(r/l)) \qquad \text{(S9)}$$

Acting analogously for the phase of the oscillations, we obtain the phase jump across the boundary:

$$\delta\varphi = \arg(1 - R_{\mathrm{gb}}\frac{k}{l}\Theta(r/l)) \approx -R_{\mathrm{gb}}\frac{k}{l}\mathrm{Im}(\Theta(r/l)) \qquad \text{(S10)}$$

and

$$R_{\text{gb}} \approx \frac{l}{k}\delta\varphi/\text{Im}(\Theta(r/l)) \tag{S11}$$

where $\text{Im}\big(\Theta(r/l)\big)$ is the imaginary parts of the function $\Theta(r/l)$. We note that eqs (S9) and (S11) become eqs (14) and (17) in the main text, respectively, after replacements: $1/\text{Re}(\Theta(r/l)) \rightarrow \sqrt{2}$ and $1/\text{Im}\big(\Theta(r/l)\big) \rightarrow \sqrt{2}$. Plots of the functions $1/\text{Re}(\Theta(r/l))$ and $1/\text{Im}\big(\Theta(r/l)\big)$ are shown in Figure 5 in the main text.

If the measurement points are at a distance from the grain boundary, linearization of eq (S1) leads to the same form of the correction term as in eqs (15) and (18) in the main text:

$$R_{\text{gb}} \approx \frac{l}{k}\delta\tau_{\text{rel}}/\text{Re}(\Theta(r/l)) - \frac{\delta r}{k} \tag{S12}$$

and

$$R_{\text{gb}} \approx \frac{l}{k}\delta\varphi/\text{Im}(\Theta(r/l)) - \frac{\delta r}{k} \tag{S13}$$

where $\delta r$ is the radial distance between measurement points. Equations (S12) and (S13) are, respectively, eqs (19) and (20) of the main text.

## S3. Modeling of Thermal Wave Field in the Vicinity of a Grain

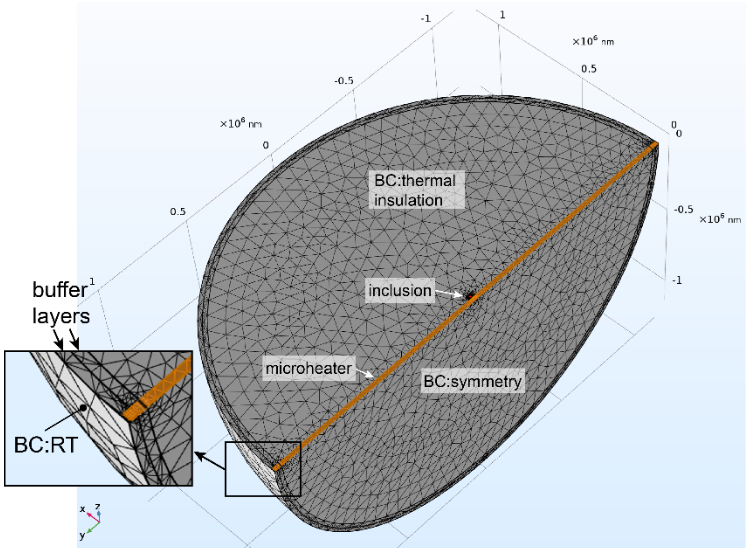


**Figure S2.** The layout of the FE model of the sample with heater and inclusion (grain). The microheater strip is yellow. The inclusion is surrounded by a dense mesh, which is seen as a black spot in the middle of the microheater strip. BC stands for Boundary Condition. RT stands for Room Temperature.

The numerical modeling of the thermal wave propagation was carried out with the use of the *Heat Transfer in Solids* interface in COMSOL Multiphysics v.5.3a (COMSOL AB) finite-elements analysis package with the preset *Thermal Perturbation, Frequency Domain* solver sequence setup.

The layout of the model is shown in Figure S2. The radius of the model is 1 mm. Properties of the STNO matrix were set to values determined by our measurements, as described in the main text. The boundary condition at the outer boundary of the model is room temperature. To model the heat flux to the environment through the bulk STNO ceramic sample embedded in epoxy (whose primary purpose was to hold the sample during polishing), the FE model had two layers of materials with artificial properties ("buffer" materials in Figure S2). The thickness of the layers was the same and equal to 20 μm. The outermost layer was introduced into the model to account for the epoxy bed, and the next layer accounted for the STNO bulk outside the 1 mm-radius included in the model. The thermal conductivity and specific thermal capacity of the buffer materials were recalculated based on the geometrical sizes of the STNO sample and its epoxy bed to have thermal resistance and thermal diffusion time approximately equal to those of the bulk STNO sample and its epoxy bed. The thermal conductivity and thermal capacity of Cr and $SiO_2$ in the microheater strip parts on top of the fake material layers were also modified to account for the physical length of the heater strip. We have verified that any further increase of the model size does not lead to a change in results in the vicinity of the inclusion.

The central part of the model, with the semispherical inclusion modeling a grain, is shown in the inset of Figure 6c of the main text. The inclusion radius was varied between 2 μm and 5 μm to investigate the grain size effect. When varying the radius of the grain, the position of the grain center was shifted to keep the distance between the grain boundary and the heater stripe edge the same for all grain sizes.

The vertical structure of the heater strip was analogous to that used in the experiment (as shown in Figure 4 of the main text). In the numerical model, the volume of the Cr film of the heater strip serves as a heat source with an evenly distributed power density corresponding to Joule power in the heater strip carrying an AC current of a 75 mA amplitude at a frequency of 100 Hz. The distributed heat source was set with stationary (time-independent) and sinusoidally oscillating with a frequency $f = 200$ Hz components. The stationery and time-dependent temperature distributions in the sample were calculated separately. The stationary temperature distribution due to the time-independent component of the Joule heat was calculated first as the linearization point. After that, the oscillating temperature field (thermal waves) was calculated as a perturbation due to the oscillating component of the Joule heat in the strip.

The thermal properties of the Cr layer and its insulating $SiO_2$ sublayer in the model were taken from the COMSOL material library. The width of the strip and the electrical conductivity of Cr layer of the microheater were set to reproduce in the model the average temperature as was measured in the experiment

with a calibrated SThM probe. The probe was calibrated with the help of a commercial PT100 resistance temperature detector (RTD) using the null-point method described in ref 3. The width of the strip in the model was equal to 1/3 of the width in the experimental setup, which is reasonable taking into account the sample porosity (as obvious from the SEM image in Figure S1 and from Figure 4 of the main text). In the experiments, strips narrower than 75 μm showed open circuits due to local pore agglomerations, which broke the strip integrity across its width (an example is seen in Figure S1b). When calculating the Joule heat power density in the Cr layer of the microheater, we assume that the Cr electrical conductivity is reduced compared to its bulk value by a factor of 0.6, which is reasonable for a thin film.

Results of numerical modeling are presented in Figure 6 of the main text as well as in Figures S3 and S4. Modification of the model to account for a pore at the grain boundary is presented in Figure S5 with a simulated thermal wave amplitude map for this model in Figure S6b.

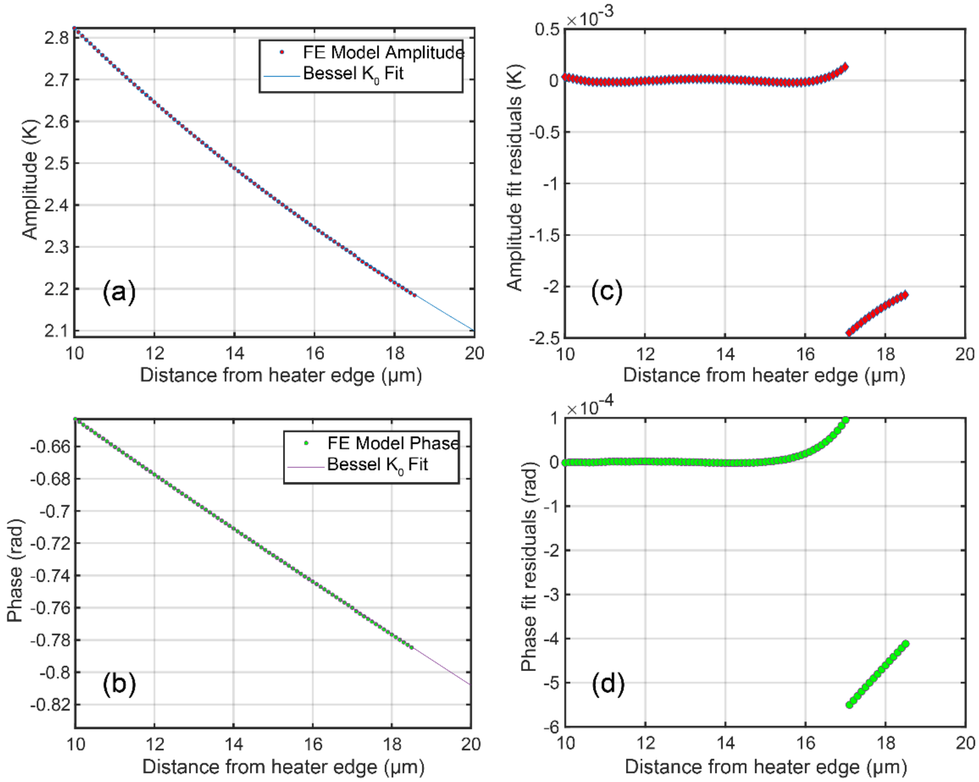


**Figure S3.** Results of the FE modeling for a grain with a radius of 4 μm, heater-boundary distance of $d_{\mathrm{hb}} = 17$ μm, and grain boundary thermal resistance of $R_{\mathrm{gb}} = 5 \times 10^{-9}$ K m$^2$ W$^{-1}$. (a) Amplitude and (b) phase as functions of the distance from the heater strip edge. Symbols are FE-calculated values, and solid lines are fits of eqs (21) and (22) of the main text to the calculated values of, respectively, (a) and (b) outside of the inclusion. (c) and (d) are fit residuals for the amplitude and phase, respectively. The values of fitting parameters are: for amplitude - $d_0 = -8.13$ μm, $l =$ 56.0 μm, and $\tau_0 = 1.93$ K, for phase - $d_0 = -3.24$ μm, $l = 50.5$ μm, and $\varphi_0 = -0.17$ rad.

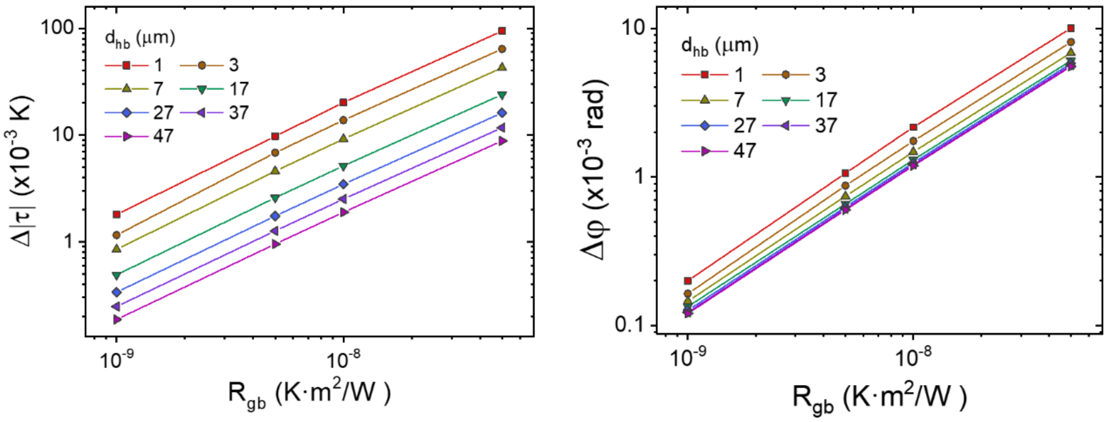


**Figure S4.** FE-model-calculated values of jumps of the thermal wave amplitude, $\Delta|\tau|$, (left) and phase, $\Delta\ \varphi$, (right) at the grain boundary as functions of the grain boundary thermal resistance, $R_{\mathrm{gb}}$, for different distances between the grain boundary and the microheater edge, $d_{\mathrm{hb}}$. The distances are indicated in the plot legends. Note that the phase jumps are significantly less dependent on the distance than the amplitude jumps.

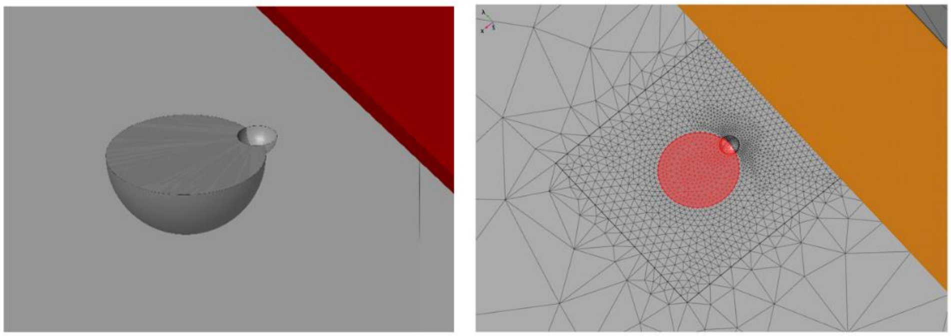

**Figure S5.** Left: Modification of the FE model, where a pore was introduced at the grain (inclusion) boundary. Right: Mesh on the model surface in and around the grain with a pore.

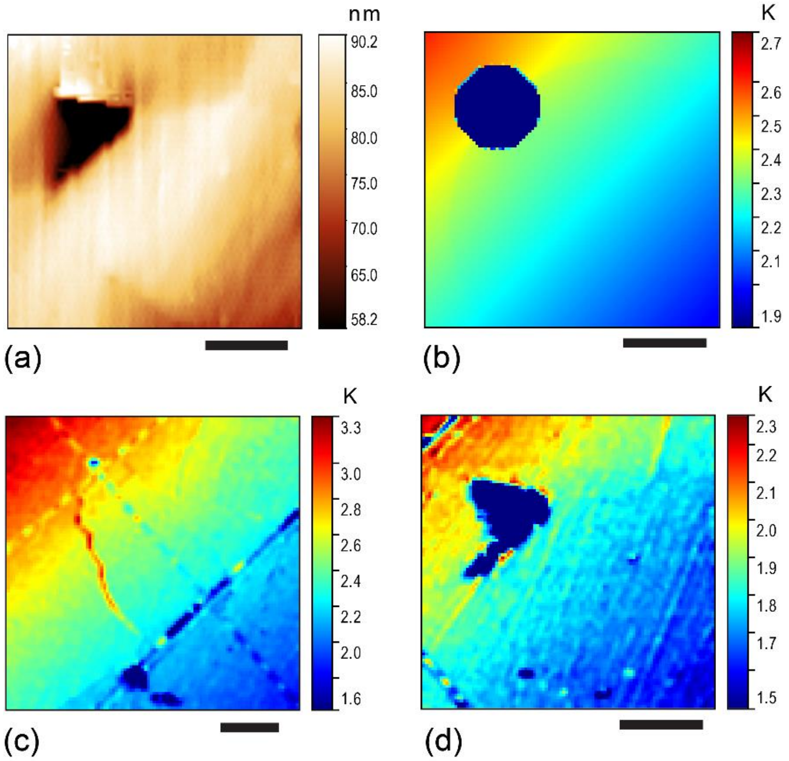


**Figure S6.** (a) A topographic map of the same area as in (d) (as well as in the maps in Figures 9a and b of the main text), the topography image was acquired in the tapping mode with the same SThM probe as the STWM maps. (b) A raw, unleveled, map simulated with an FE model imitating the sample in (a) and (d). The map corresponds to the map in Figure 9c of the main text. The model of the grain is shown in Figure S5. (c) Unleveled map of the probe signal amplitude, same as in Figure 8a of the main text, which was smoothed with a 2 px-wide Gaussian filter. The values of the map were recalculated into temperature assuming the probe-sample boundary thermal resistance $R_{\mathrm{bs}}^{\mathrm{th}} = 2 \times 10^7$ K W$^{-1}$ (see Section S5 and plot in Figure S8a below). With this value of the boundary resistance, the simulated and experimental STWM maps could be reasonably well matched. This value is about two times larger than $R_{\mathrm{bs}}^{\mathrm{th}}$ for ceramic samples in our earlier publication, ref 3. (d) Same as in panel (c) but corresponding to the map in Figure 9a of the main text. The scale bars in all panels are 2 µm.

## S4. Characterization of the Noise of Electronics and Estimation of Sensitivity

The noise power density spectrum at the output of the preamplifier (with a fully assembled and powered measurement circuit, a 0.25 mA DC current flowing through the probe installed in the balanced Wheatstone bridge and out of contact with a sample) was measured with the use National Instruments NI 9222 AI (Analogue Input) module installed in a cDAQ-9191 CompactDAQ Chassis (National Instruments, USA). The data acquisition was controlled with the help of a LabView program, which, in particular, performed a Fast Fourier Transform (FFT) of the time-dependent signal acquired by the AI module to obtain the voltage noise power density spectrum. The measurement yielded 35 µV/Hz$^{1/2}$ at the output of the

preamplifier in the frequency range around the frequency of 200 Hz used in the thermal wave measurements. With the lock-in time constant of 10 ms and the 4th-order low-pass filter set in the experiments, the lock-in bandwidth is 7.8 Hz,[4] and we obtain the voltage noise of 98 μV RMS (which agreed well with the noise level measured with the lock-in at its input). With the account of averaging over 100 measurement points at each pixel of a thermal wave map, the RMS voltage noise is further reduced by a factor of 10 to 9.8 μV RMS. Next, we take into account that for the dispersions of the measured values in eq (23) of the main text, the following holds:

$$\sigma(\delta\tau_{\mathrm{rel}}) = \sigma\left(\frac{|\tau_1| - |\tau_2|}{|\tau_1|}\right) = \sigma(\varphi_2 - \varphi_1) \approx \sqrt{2}\frac{\sigma(|\tau|)}{|\tau|},$$

where $\sigma$ means "dispersion", and $\sigma(|\tau|)$ is equal to the RMS of the signal noise. The expression above was derived with the use of basic properties of statistical variance, which is $\sigma^2$, and application of the standard delta method with the account that $\sigma(|\tau|) \ll |\tau|$. With $\sigma(|\tau|) = 9.8$ μV and $|\tau| \approx 15$ mV as at the grain boundary in the map in Figure 8a of the main text, we get $\sigma(\tau_{\mathrm{rel}}) = 9.2 \times 10^{-4}$. Substitution of this value for $\delta\tau_{\mathrm{rel}}$ in eq (23) of the main text with $h_\tau = 0.6$, yields $R_{\mathrm{gb}} = 3.4 \times 10^{-9}$ K m$^2$ W$^{-1}$, which can be viewed as the minimal detectable $R_{\mathrm{gb}}$ with the used settings. Apparently, this value can be further reduced, for instance, by averaging over a larger number of datapoints and increasing the probe current used to measure probe resistance, which increases the signal-to-noise ratio.

## S5. Modeling of Dynamic Response of the KNT Probe

To achieve an optimal performance in measurement with thermal waves, it is critical to know the dynamic-signal properties of the SThM probe. With the help of an FE model of the probe, we are able to reveal different dynamic aspects of the probe response to sample temperature variations. We performed numerical experiments with a calibrated probe model by varying the thermal resistance of the boundary between the probe apex and a sample, sweeping the sample temperature (in a range between 40 °C and 120 °C) and calculating the resulting probe resistance variations.

Numerical modeling of the KNT-SThM-2an (KNT) probe was carried out with the use of the *Joule Heating* interface of COMSOL Multiphysics finite elements analysis package with the preset *Thermal Perturbation, Frequency Domain* solver sequence setup. The model layout with boundary conditions is shown in Figure S7. The model included only the cantilever, without the probe chip, and was nearly identical to that used in our previous publication, ref 3. The dimensions and thickness of the cantilever and the gold electrical leads were taken from the manufacturer's specifications (Kelvin Nanotechnology, UK), and the dimensions of the Pd sensor stripe were determined from our own scanning electron microscopy

(SEM) images. The thickness of the Pd stripe was set equal to 40 nm as indicated in the manufacturer's specifications. The values of heat capacity and density of materials were taken from the COMSOL material library. Other necessary material properties were a subject of the model calibration as described in ref 3. Since the probe used for this work was from the same batch (box) as in ref 3, the probe parameters in the model are nearly identical to those in ref 3.

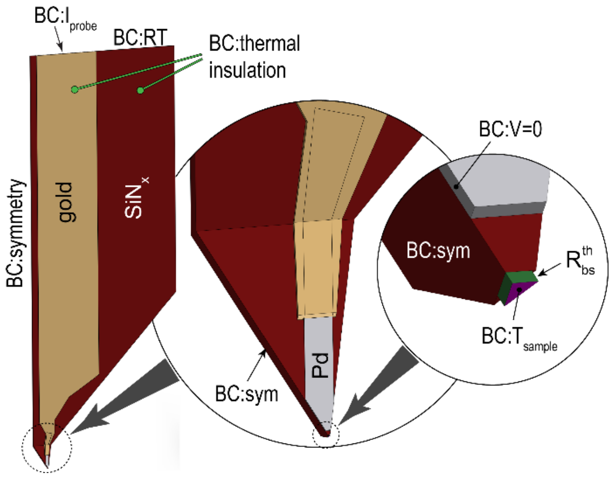


**Figure S7.** Layout of the FE model of the KNT probe. BC stands for Boundary Condition, RT stands for Room Temperature, and "sym" stands for Symmetry. Only half of the cantilever is included in the model owing to the Symmetry boundary conditions along the cut-plane faces. A DC current $I_{\mathrm{probe}}$ is injected on the chip-side face of the gold lead, as indicated by the arrow. Room temperature boundary condition is set on the chip-side face of the $SiN_x$ cantilever and the gold lead. The boundary condition on all external boundaries, except those indicated, is thermal insulation.

After calibration, the Pd and gold thermal and electrical conductivities in the model were reduced to 27% of their bulk values (provided in COMSOL's material library). The rationale behind this reduction is explained in detail in ref 3. The Pd conductivity was calculated as $\sigma_{\mathrm{Pd}} = \sigma_0/(1 + \alpha_{\mathrm{Pd}}\Delta T)$, where $\sigma_0$ is the conductivity at room temperature. The thermal coefficient of resistance (TCR) of Pd was determined to be equal to $\alpha_{\mathrm{Pd}} = 1.16 \times 10^{-3}$ 1/K. The thermal conductivity of the cantilever materials, $SiN_x$, was set to $k_{\mathrm{SiN}} = 3.2$ W/(m·K).

A sample in contact with the probe was modeled through a boundary condition "Temperature" on the outer boundary (face) at the probe apex contact area, which is indicated as BC:$T_{\mathrm{sample}}$ in Figure S7. To model the sum of a sample thermal spreading resistance and the probe-sample boundary resistance, $R_{\mathrm{bs}}^{\mathrm{th}}$, a small domain of a material with a total thermal resistance equal to $R_{\mathrm{bs}}^{\mathrm{th}}$ was introduced between the probe contact area and the face with BC:$T_{\mathrm{sample}}$ as shown in Figure S7. To sweep $R_{\mathrm{bs}}^{\mathrm{th}}$, the thermal conductivity of

the material in this domain was swept. The $R_{\mathrm{bs}}^{\mathrm{th}}$ defined this way is the parameter in the plots in Figure S8 below.

The stationery and time-dependent temperature distributions in the probe were calculated separately. The stationary temperature distribution due to the time-independent average sample temperature was calculated first as the linearization point. The stationary component of $T_{\mathrm{sample}}$ was varied from 40 °C to 140 °C. The oscillating temperature field was calculated as a perturbation due to the time-dependent, harmonically oscillating component of $T_{\mathrm{sample}}$; the oscillation amplitude was 1 K.

The probe response to the sample temperature change is the change of the probe total electrical resistance. The electrical resistance of the probe, $R_{\mathrm{probe}}$, was calculated simultaneously with the temperature field in the probe with the use of the *Electric Current* component of the COMSOL's *Joule Heating* interface. For that, a DC current $I_{\mathrm{probe}} = 0.25$ mA was injected in the gold lead as a boundary condition on the chip-side face of the gold lead, as indicated in Figure S7. The probe response is calculated as the voltage appearing on the gold lead boundary, where the probe current is injected. This voltage is used to calculate the probe electrical resistance $R_{\mathrm{probe}}$.

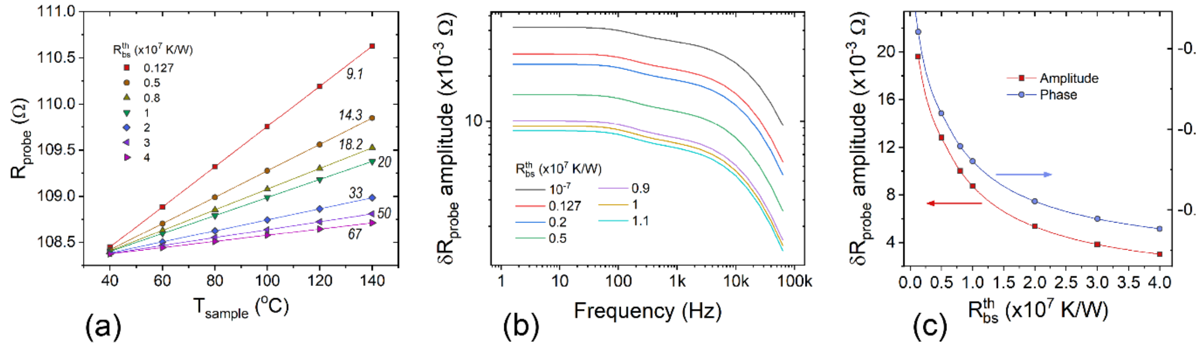


**Figure S8.** (a) Probe electrical resistance, $R_{\mathrm{probe}}$, as functions of the stationary, average component of sample temperature, $T_{\mathrm{sample}}$, for different values of the probe-sample contact interface thermal resistance, $R_{\mathrm{bs}}^{\mathrm{th}}$. Symbols are calculated values, and lines are linear fits. Numbers at the curves illustrate how strongly the probe sensitivity is reduced as a result of probe-sample boundary thermal resistance. See text for details. (b) The magnitude of the probe response as a function of the frequency of sample temperature oscillations for several values of probe-sample boundary thermal resistance, $R_{\mathrm{bs}}^{\mathrm{th}}$. (c) Simulated amplitude and phase of probe electrical resistance as functions of the probe-sample boundary thermal resistance, $R_{\mathrm{bs}}^{\mathrm{th}}$. The frequency of the sample temperature oscillations was 200 Hz.

Figure S8a show $R_{\mathrm{probe}}$ as functions of the stationary $T_{\mathrm{sample}}$ for different $R_{\mathrm{bs}}^{\mathrm{th}}$. The change of the sample temperature can be determined from the change of probe resistance as:

$$\Delta T_{\mathrm{sample}} = \frac{\Delta R_{\mathrm{probe}}}{\alpha' R_0},$$

where $R_0$ is the room temperature resistance of the sensor, $\Delta R_{\text{probe}}$ is the probe resistance change, and $\alpha'$ is a coefficient introduced analogously to the TCR of the Pd thermoresistive element. The numbers at the curves in Figure S8a indicate values of the ratio of $\alpha_{\text{Pd}}/\alpha'$, which can be deduced from the slopes of the corresponding lines. In other words, these numbers are the dividers that need to be applied to the TCR of the probe thermoresistor to convert it into TCR with respect to the sample temperature. As expected, the sample-temperature-referenced TCR of the probe drops with increasing probe-sample thermal boundary resistance.

Figure S8b shows the magnitude of the probe response as a function of the frequency of sample temperature oscillations (Bode plots) for several values of probe-sample boundary thermal resistance, $R_{\text{bs}}^{\text{th}}$. As expected, the probe response strongly depends on $R_{\text{bs}}^{\text{th}}$. However, it should be stressed that the strong dependence exists in the magnitude but not in the shape of the frequency response, which is weekly dependent on $R_{\text{bs}}^{\text{th}}$. The cut-off frequency, which we define as the frequency at which the response magnitude is one-half of the response at low frequency, is about 10 kHz for all the $R_{\text{bs}}^{\text{th}}$ values used in the modeling.

Figure S8c displays the simulated amplitude and phase of probe electrical resistance as functions of the probe-sample boundary thermal resistance, $R_{\text{bs}}^{\text{th}}$, for the $R_{\text{bs}}^{\text{th}}$ values close to $1 \times 10^7$ K W$^{-1}$. The frequency of the sample temperature oscillations was 200 Hz in these calculations. The stationary component of the sample temperature was 50 °C; however, results at different average sample temperatures in the range from 40 °C to 140 °C were indistinguishable.

## References


(1) Carslaw, H. S.; Jaeger, J. C. Conduction of Heat in Solids. Clarendon Press: 1959; Chapter 7.

(2) Abramowitz, M.; Stegun, I. A. *Handbook of Mathematical Functions: With Formulas, Graphs, and Mathematical Tables*, Dover Publications: 1965.

(3) Alikin, D.; Zakharchuk, K.; Xie, W.; Romanyuk, K.; Pereira, M. J.; Arias-Serrano, B. I.; Weidenkaff, A.; Kholkin, A.; Kovalevsky, A. V.; Tselev, A. Quantitative Characterization of Local Thermal Properties in Thermoelectric Ceramics Using "Jumping-Mode" Scanning Thermal Microscopy. *Small Methods* **2023,** *7* (4), 2201516.

(4) HF2 User Manual. 50 MHz Lock-in Amplifier. Release 24.01 ed.; Zurich Instruments AG: 2023.
